# Integrated Assessment of the Functional Activity of the Gut Environment and Metabolic Profiles in Rats Fed an Insect-Based (*Tenebrio molitor*) Diet

**DOI:** 10.3390/ijms27177627

**Published:** 2026-08-26

**Authors:** Remigiusz Gałęcki, Adriana Nowak, Joanna Nizioł, Tomasz Ruman, Aneta Płaza-Altamer, Justyna Szulc

**Affiliations:** 1 Department of Veterinary Prevention and Feed Hygiene, Faculty of Veterinary Medicine, University of Warmia and Mazury in Olsztyn, Oczapowskiego 13, 10-719 Olsztyn, Poland; 2 Department of Environmental Biotechnology, Faculty of Biotechnology and Food Sciences, Lodz University of Technology, Wólczańska 171/173, 90-530 Łódź, Poland; 3 Department of Polymers and Biopolymers, Faculty of Chemistry, Rzeszów University of Technology, al. Powstańców Warszawy 12, 35-959 Rzeszów, Poland; 4 Department of Inorganic and Analytical Chemistry, Faculty of Chemistry, Rzeszów University of Technology, al. Powstańców Warszawy 12, 35-959 Rzeszów, Poland

**Keywords:** model organisms, metabolome analysis, spleen, liver, nutrition, yellow mealworm, novel food, fecal enzyme activity, short-chain fatty acids, fecal water genotoxicity

## Abstract

Edible insects are increasingly proposed as sustainable protein sources in animal nutrition, yet their effects on gut microbial function, intestinal safety, and systemic metabolism require comprehensive evaluation. This study investigated the impact of a diet containing 35% *Tenebrio molitor* meal on the functional activity of gut environment and host metabolic profiles in rats, in comparison with diets containing chicken hydrolysate or a standard laboratory diet. Female Wistar rats were fed the experimental diets for 60 days, after which fecal short-chain fatty acids (SCFAs), microbial enzyme activity, fecal water genotoxicity, and liver and spleen metabolomes were assessed. Rats fed the *T. molitor* diet exhibited significantly lower fecal concentrations of major SCFAs, including acetate, butyrate, valerate, and branched-chain fatty acids (BCFAs), indicating altered colonic fermentation. Both β-glucosidase and β-glucuronidase activities differed significantly between groups and were highest in the *T. molitor* group. β-Glucuronidase activity also decreased over time, whereas no significant group vs. time interaction was observed. Fecal water genotoxicity decreased markedly in the *T. molitor* group over time. Untargeted UHPLC–HRMS metabolomics revealed pronounced diet-dependent differences in metabolite profiles. In the liver, the insect-based diet was associated with the enrichment of glycerophospholipid metabolism, primary bile acid biosynthesis, and pyrimidine metabolism pathways, as well as lower abundance of metabolites assigned to riboflavin- and phenylalanine-related pathways. In the spleen, diet-associated differences involved metabolites assigned to lipid-related pathways, including glycerophospholipid, linoleic acid, and steroid-related metabolism, as well as nucleotide-associated pathways. These findings support further evaluation of complete insect-based formulations and highlight specific metabolic pathways that should be considered when evaluating the safety and physiological implications of insect-based feeds.

## 1. Introduction

Insects have emerged as a sustainable and nutritious source of food for both humans and animals. They require minimal land and water resources and can be reared on organic side-streams, effectively turning them into high-quality nutrients [1,2]. Many insect species are rich in protein, lipids, vitamins, and minerals, and serve as natural food sources for many animal species [1,2,3]. Numerous studies have demonstrated that insects have a favorable nutritional composition, making them attractive feed components [4,5,6,7]. Insects can be produced in a circular economy model, contributing to sustainable animal husbandry [8,9,10]. Edible insects can also be used in the development of veterinary diets for companion animals [11].

In particular, the yellow mealworm (*Tenebrio molitor*) has been highlighted for its potential use as food and feed. Mealworm larvae typically contain about 50–60% crude protein (dry basis) with a rich amino acid profile, and substantial amounts of fat rich in unsaturated fatty acids [12,13,14]. *Tenebrio molitor* also provides fiber in the form of chitin as well as micronutrients (B vitamins, minerals) with health-promoting properties [15,16,17]. These qualities make *T. molitor* a promising feed ingredient for monogastric animals (poultry, pigs, fish, dogs, cats), and studies have shown that it can fully replace conventional protein sources such as soybean meal or fishmeal without compromising growth performance, while also modulating gut morphology, microbiota, and immune indicators [18,19,20,21,22,23].

The laboratory rat (*Rattus norvegicus*) is a well-established model for studying nutrition, gut microbiology, and systemic metabolism. Rats share key gastrointestinal and metabolic features with other mammals, and their gut environment responds predictably to dietary changes, making them suitable for investigating host-microbe interactions [24,25,26]. The larger size and well-characterized physiology of rats also enable detailed metabolic and immunological measurements, providing a relevant preclinical platform for assessing novel diets.

Short-chain fatty acids (SCFAs) are the major end-products of microbial fermentation in the colon. They serve as an energy source for colonic epithelial cells and exert regulatory effects on gut barrier function, inflammation, and systemic metabolism [27]. Therefore, fecal SCFA levels provide an integrative readout of colonic fermentation and host–microbiota metabolic interactions [28,29]. Similarly, the collective activity of gut microbial enzymes, such as β-glucosidase and β-glucuronidase, reflects functional shifts in the microbiome that may affect nutrient processing and the generation of harmful metabolites [30]. Fecal water genotoxicity is another marker of colonic health, capturing the net balance of pro- and anti-genotoxic factors in the gut lumen [31,32,33].

Beyond the gut, the metabolomic profiling of host organs can reveal the systemic effects of diet–microbiota interactions [34]. As a key immune organ, the spleen can provide insight into diet-induced changes in immunometabolism [35,36,37]. These complementary approaches enable a multi-parameter assessment of how insect diets affect both intestinal function and host physiology.

Despite growing interest in insect-based feeds, comprehensive evaluations of their effects in mammals remain limited [38,39,40,41]. Few studies have simultaneously assessed the effects of high-inclusion insect diets using gut-derived functional biomarkers (SCFAs, fecal enzymes, genotoxicity) alongside systemic metabolomics endpoints. In particular, data on fecal water genotoxicity in response to insect-based diets are lacking, and the systemic metabolomic effects of consuming insect meals, as measured by organ metabolomics, remain poorly characterized [42,43]. These gaps underscore the need for an integrative assessment to evaluate both the potential benefits and risks of insect-based diets in a monogastric model.

The aim of this study was to assess the impact of a diet containing 35% *T. molitor* meal on SCFA levels, fecal enzyme activity, fecal water genotoxicity, and liver and spleen metabolomics in rats, compared with a chicken hydrolysate diet and a standard laboratory diet. It was hypothesized that rats fed the *T. molitor*-based diet would exhibit significant differences in these parameters relative to rats fed chicken hydrolysate or a standard diet, reflecting the unique nutritional and bioactive composition of insect meal. The present study employs an integrated, multi-parameter approach that, for the first time, combines the above analyses. Notably, this work provides the first evaluation of fecal water genotoxicity following *T. molitor* consumption in mammals. The spleen was selected as a complementary tissue because nutritional interventions can alter both host-derived metabolites and the metabolic activity of the gut microbiota. Circulating microbial products and metabolites may affect splenic macrophages, dendritic cells, and lymphocyte populations, thereby influencing systemic immune tone. The spleen is also functionally linked to the gut and liver through vascular, lymphatic, neural, and immunological communication routes, forming a broader gut-spleen-liver axis. To the best of our knowledge, this is the first metabolomic characterization of splenic tissue in response to an insect-based diet.

## 2. Results

### 2.1. Analysis of Fecal Enzymes and Short-Chain Fatty Acids

**Figure 1:**
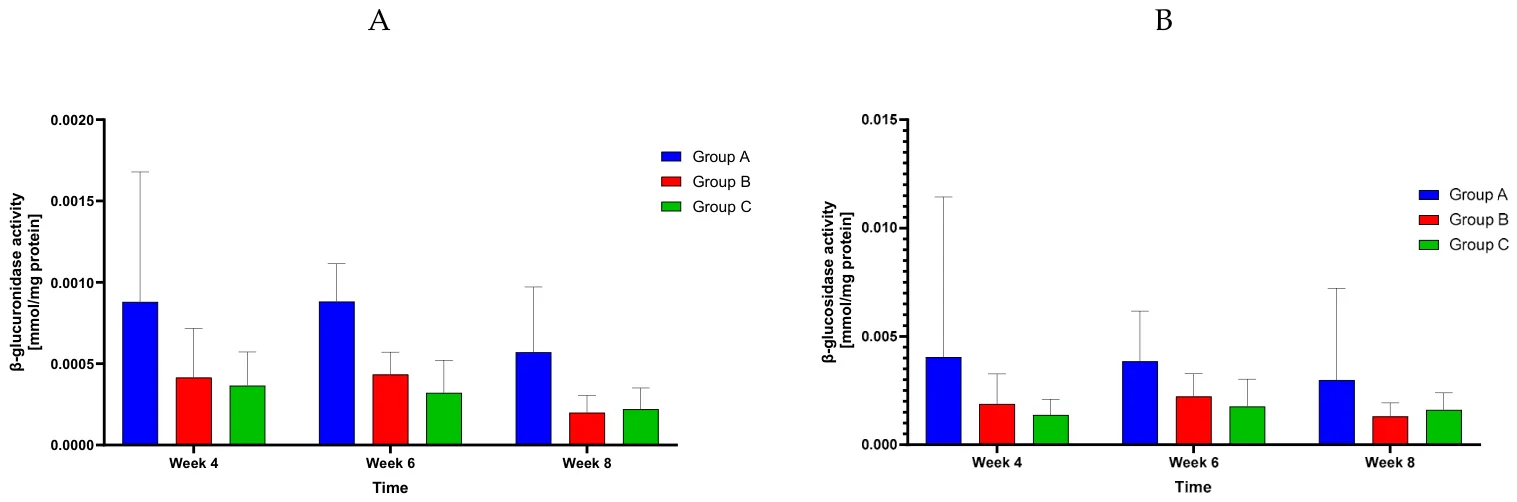
Temporal changes in (**A**) β-glucuronidase and (**B**) β-glucosidase activity in fecal samples collected from rats fed different diets. Legend: Groups: A (35% *T. molitor* meal), B (chicken hydrolysate diet), C (standard laboratory rat diet).

β-glucuronidase activity in rat feces generally decreased over the 8-week experimental period and differed significantly between dietary groups. Mean activity in Group A (35% *T. molitor*) ranged from 0.000571 to 0.000883 mmol/mg protein compared with 0.000200–0.000434 mmol/mg protein in Group B and 0.000221–0.000366 mmol/mg protein in Group C. Repeated-measures ANOVA revealed a significant effect of time (df = 2, F = 18.16, *p* < 0.0001, ηp^2^ = 0.32), and group (df = 2, F = 78.62, *p* < 0.0001, ηp^2^ = 0.79), whereas the group vs. time interaction was not significant (df = 4, F = 1.22, *p* = 0.302, ηp^2^ = 0.059). Activity was consistently highest in Group A (Figure 1A).

β-glucosidase activity differed between the dietary groups, but it remained relatively stable throughout the experimental period. In Group A, mean activity decreased slightly from 0.00405 to 0.00299 mmol/mg protein while lower values were observed in Group B (0.00189–0.00131 mmol/mg protein) and Group C (0.00138–0.00162 mmol/mg protein). Repeated-measures ANOVA demonstrated a significant main effect of group on β-glucosidase activity (df = 2, F = 22.57, *p* < 0.001, ηp^2^ = 0.52), whereas neither time (df = 2, F = 1.48, *p* = 0.230, ηp^2^ = 0.034) nor the group vs. time interaction (df = 4, F = 0.52, *p* = 0.722, ηp^2^ = 0.024) was significant. The highest values were observed in rats fed the *T. molitor*–based diet (Figure 1B).

Across the 8-week feeding period, SCFA concentrations differed between the dietary treatments and, for several acids, changed over time. Formic acid concentrations remained lowest in Group A (11.71–17.17 mg/g) compared with Group B (24.02–39.36 mg/g) and Group C (19.48–31.12 mg/g). Significant effects of group (df = 2, F = 23.43, *p* < 0.000001, ηp^2^ = 0.53) and time (df = 2, F = 8.44, *p* = 0.000456, ηp^2^ = 0.16) were observed, with no significant group vs. time interaction.

Acetic acid followed a similar pattern, with lower concentrations in Group A (12.17–15.97 mg/g) than in Group B (31.58–49.03 mg/g) and Group C (15.85–31.92 mg/g). Both group (df = 2, F = 25.13, *p* < 0.000001, ηp^2^ = 0.54) and time had significant effects (df = 2, F = 6.54, *p* = 0.002299, ηp^2^ = 0.13), whereas the group vs. time interaction was not significant.

Propionic acid increased over time across groups, although variability was high (Group A: 8.27–15.69 mg/g; Group B: 13.58–20.60 mg/g; Group C: 9.16–23.43 mg/g). A significant effect of time was observed (df = 2, F = 3.70, *p* = 0.02884, ηp^2^ = 0.081), whereas the effects of group and group vs. time interaction were not significant.

Butyric acid concentrations increased markedly over time, particularly in Group B (15.75–95.57 mg/g), compared with Group A (11.38–26.06 mg/g) and Group C (19.18 ± −66.43 mg/g). Repeated-measures ANOVA showed significant effects of group (df = 2, F = 31.94, *p* < 0.000001, ηp^2^ = 0.60) and time (df = 2, F = 13.11, *p* = 0.000011, ηp^2^ = 0.24), and group vs. time interaction (df = 4, F = 3.34, *p* = 0.01375, ηp^2^ = 0.14), indicating diet-dependent changes in butyrate concentrations.

Branched-chain fatty acids (BCFAs) and related acids exhibited greater variability. Isobutyric acid remained low in Group A (3.41–5.31 mg/g), with higher values in Group B (15.83–20.47 mg/g), and considerable variability in Group C (7.28–19.85 mg/g). No significant effects of group, time, or their interaction were observed.

Isovaleric acid concentrations were also lowest in Group A (8.72–11.46 mg/g) and higher in Groups B (18.11–43.48 mg/g) and C (18.27–34.43 mg/g). A significant effect of group was observed (df = 2, F = 7.92, *p* = 0.001208, ηp^2^ = 0.27), whereas time and the group vs. time interaction were not significant.

Valeric acid concentrations also differed between the dietary treatments with the lowest values in Group A (5.86–8.16 mg/g), compared with Group B (26.29–44.52 mg/g) and Group C (18.44–35.95 mg/g). Repeated-measures ANOVA confirmed a significant effect of group (df = 2, F = 11.71, *p* = 0.000091, ηp^2^ = 0.27), whereas neither time nor the group vs. time interaction were significant. Detailed data are presented in Table 1.

The strength of the associations between selected SCFAs and the sum of the remaining acids differed between the groups. For acetic acid, the interaction was significant (df = 2, F = 3.45, *p* = 0.0416). The slope in Group C was 9.51 (95% CI: 5.60–13.42), whereas lower slopes were observed in Group A (4.03, 95% CI: 2.47–5.59; A vs. C: *p* = 0.0107) and Group B (4.14, 95% CI: 2.75–5.53; B vs. C: *p* = 0.0112). A significant interaction was observed for valeric acid (df = 2, F = 3.53; *p* = 0.0390). In Group C, the slope was positive (5.01, 95% CI: 3.77–6.26), while in Group B, it was close to zero (0.26, 95% CI: −3.10–3.63) and differed significantly from that in Group C (B vs. C: *p* = 0.0094). No significant differences in slopes were observed for the remaining acids.

### 2.2. Genotoxicity Assessment of Fecal Water

**Figure 2:**
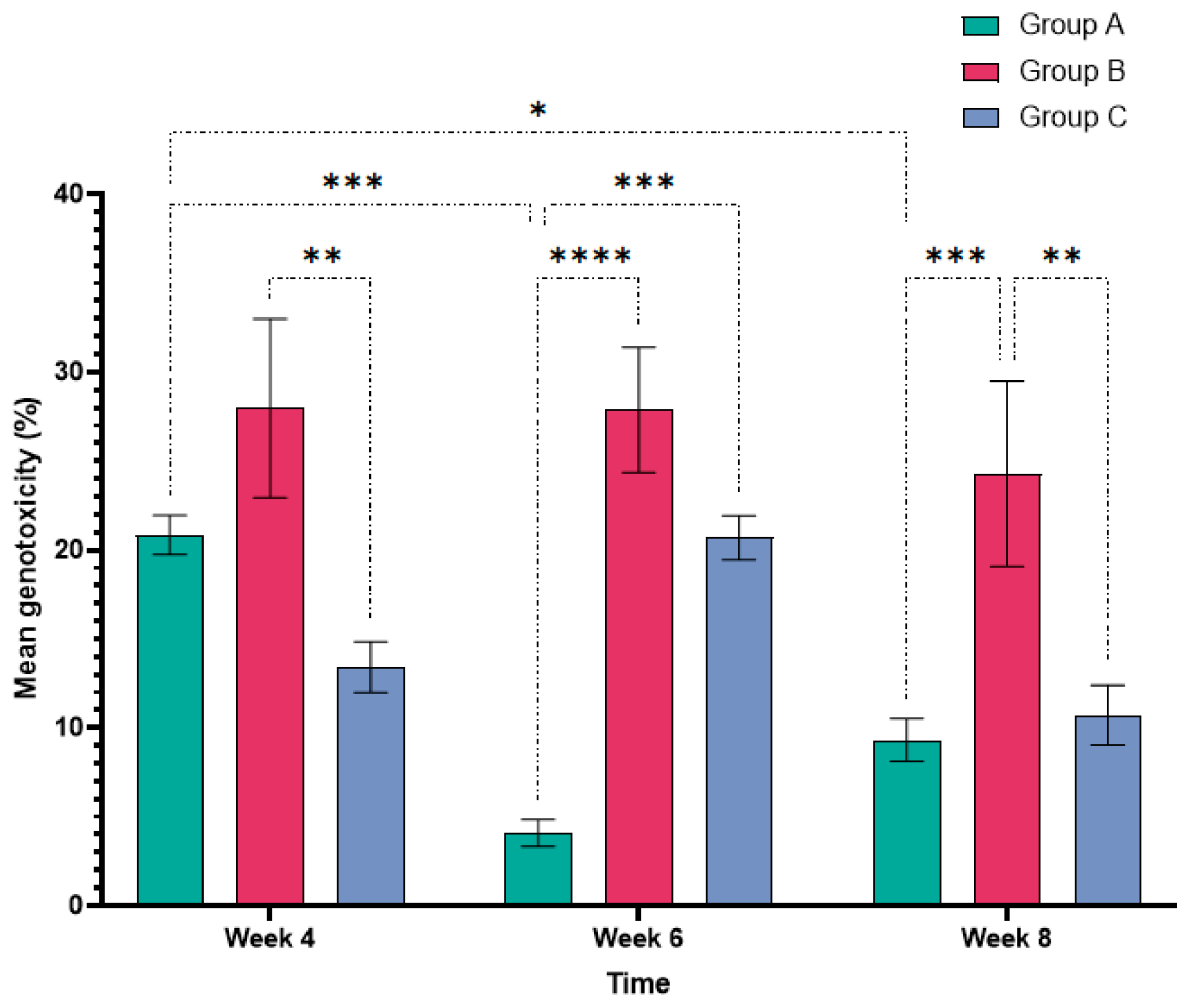
Mean genotoxicity of fecal water of rats administered different diets after 4, 6, and 8 weeks of feeding (±SEM). Legend: * *p* < 0.05; ** *p* < 0.01; *** *p* < 0.001; **** *p* < 0.0001; Groups: A (35% *T. molitor* meal), B (chicken hydrolysate diet), C (standard laboratory rat diet).

The average DNA damage in non-exposed cells in the negative control group was 1.93%. In Group A, genotoxicity decreased over time, with the greatest reduction (approximately 6-fold) occurring after 6 weeks. In Group B, fecal water genotoxicity remained relatively stable and decreased only slightly after 8 weeks of feeding, whereas Group C showed an increase after 6 weeks, followed by a slight decrease after 8 weeks. A statistically effect of group (df = 2, F = 27.40, *p* < 0.001, ηp^2^ = 0.57) and group vs. time interaction (df = 4, F = 4.37, *p* = 0.003, ηp^2^ = 0.17) were observed. At weeks 4 and 6, all groups differed significantly (*p* < 0.001), with the highest genotoxicity in Group B and the lowest genotoxicity in Group C (week 4) and Group A (week 6). At week 8, genotoxicity remained significantly higher in Group B than in Groups A and C (*p* < 0.001), whereas no differences were observed between Groups A and C. Results are presented in Figure 2. Detailed descriptive statistics are presented in the Supplementary Materials (File S1; Table S1).

Multivariable regression analysis was performed to evaluate whether SCFA concentrations and fecal enzyme activity were independently associated with genotoxicity after adjustment for dietary group. At week 4, the model was significant (R^2^ = 0.417, adjusted R^2^ = 0.222, *p* = 0.0449) although no individual predictor reached statistical significance. At week 6, the model showed the strongest explanatory power (R^2^ = 0.797, adjusted R^2^ = 0.729, *p* < 0.000001). Group remained significantly associated with genotoxicity, with both Group B and Group C showing higher genotoxicity than Group A. Among the biochemical variables, formic acid was positively associated with genotoxicity, while butyric acid showed a negative association. Fecal enzyme activity was not a significant independent predictor.

At week 8, the model remained significant (R^2^ = 0.466, adjusted R^2^ = 0.287, *p* = 0.0162). Group B showed significantly higher genotoxicity than Group A, while Group C did not differ significantly from Group A. Butyric acid was negatively associated with genotoxicity while fecal enzyme activity was not significant. Model comparisons indicated that dietary group was the dominant explanatory factor, whereas the addition of SCFA concentration and enzyme activity did not significantly improve the group-only model. Detailed regression results, including β-coefficients, standard errors, 95% confidence intervals, and individual *p*-values for each independent variable are included in Supplementary Materials (File S2).

### 2.3. Liver Metabolomics

**Figure 3:**
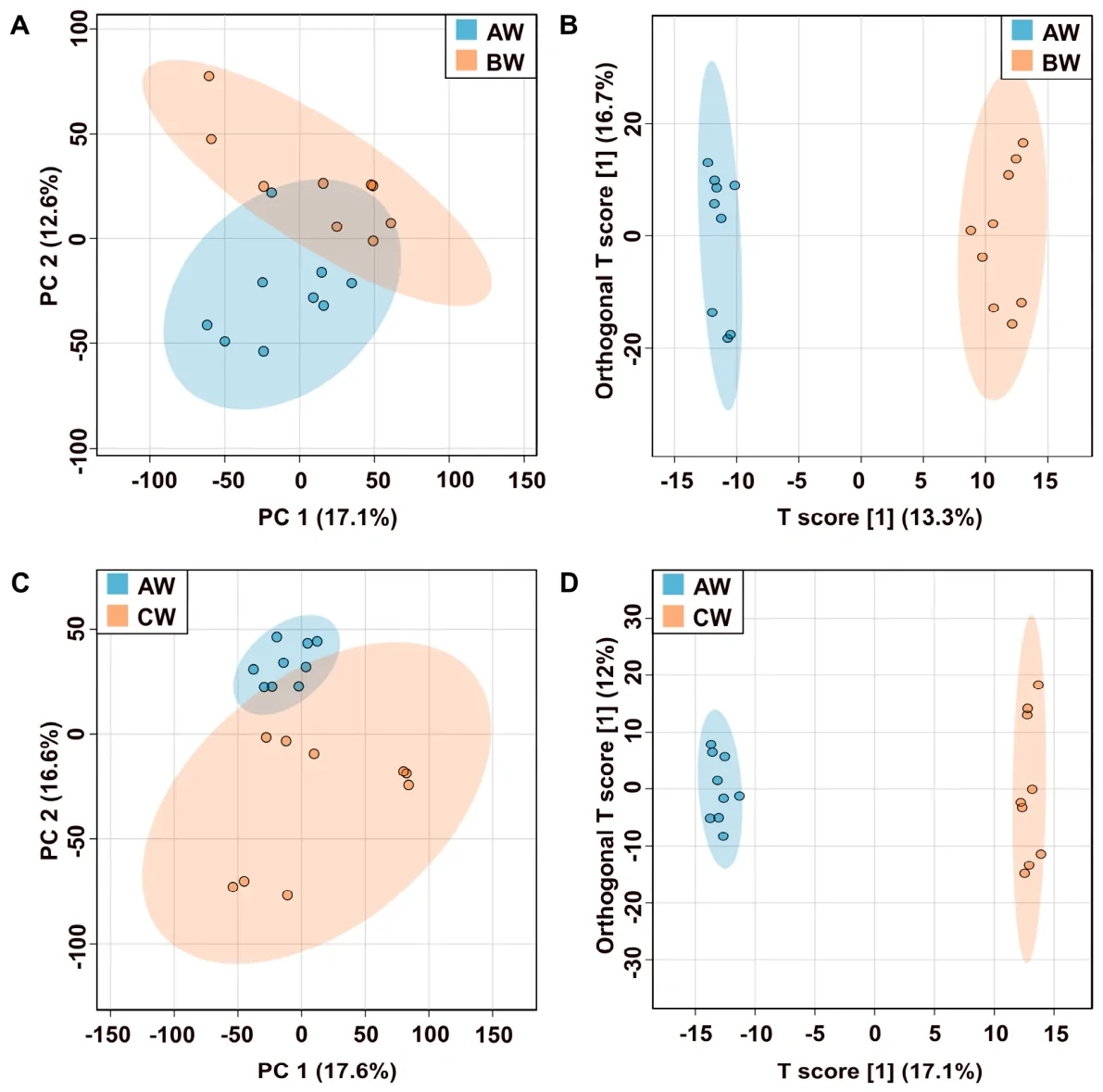
Multivariate statistical analyses of liver metabolite profiles from rats fed a diet containing 35% *T. molitor* meal (AW) compared with rats fed control diets: a diet containing chicken hydrolysate (Group BW; panels **A** and **B**) or a standard laboratory diet (Group CW; panels **C** and **D**). Legend: Each point represents an individual sample, and the 95% confidence ellipses illustrate within-group variability. (**A**,**C**) Principal component analysis (PCA) score plots. The observed clustering indicates diet-related differences in liver metabolic profiles. (**B**,**D**) Orthogonal partial least squares discriminant analysis (OPLS-DA) score plots showing separation between the dietary groups.

Unsupervised PCA of the entire UHPLC-HRMS metabolomics dataset revealed that liver samples from rats fed the 35% *T. molitor* diet (AW) tended to separate from those fed chicken hydrolysate (BW) (PC1: 17.1%, PC2: 12.6%), and clearly separated from the standard diet (CW) group (PC1: 17.6%, PC2: 16.6%) (Figure 3A,C). Distinct clustering patterns and differential overlap of the 95% confidence intervals indicated diet-dependent variation in the liver metabolome.

Supervised OPLS-DA confirmed clear and stable separation between AW and BW (predictive component: 13.3%; orthogonal component: 16.7%) and between AW and CW (17.1% and 12%, respectively) (Figure 3B,D). The minimal overlap of the 95% confidence intervals further confirmed distinct liver metabolite profiles in response to the *T. molitor*-based diet.

**Figure 4:**
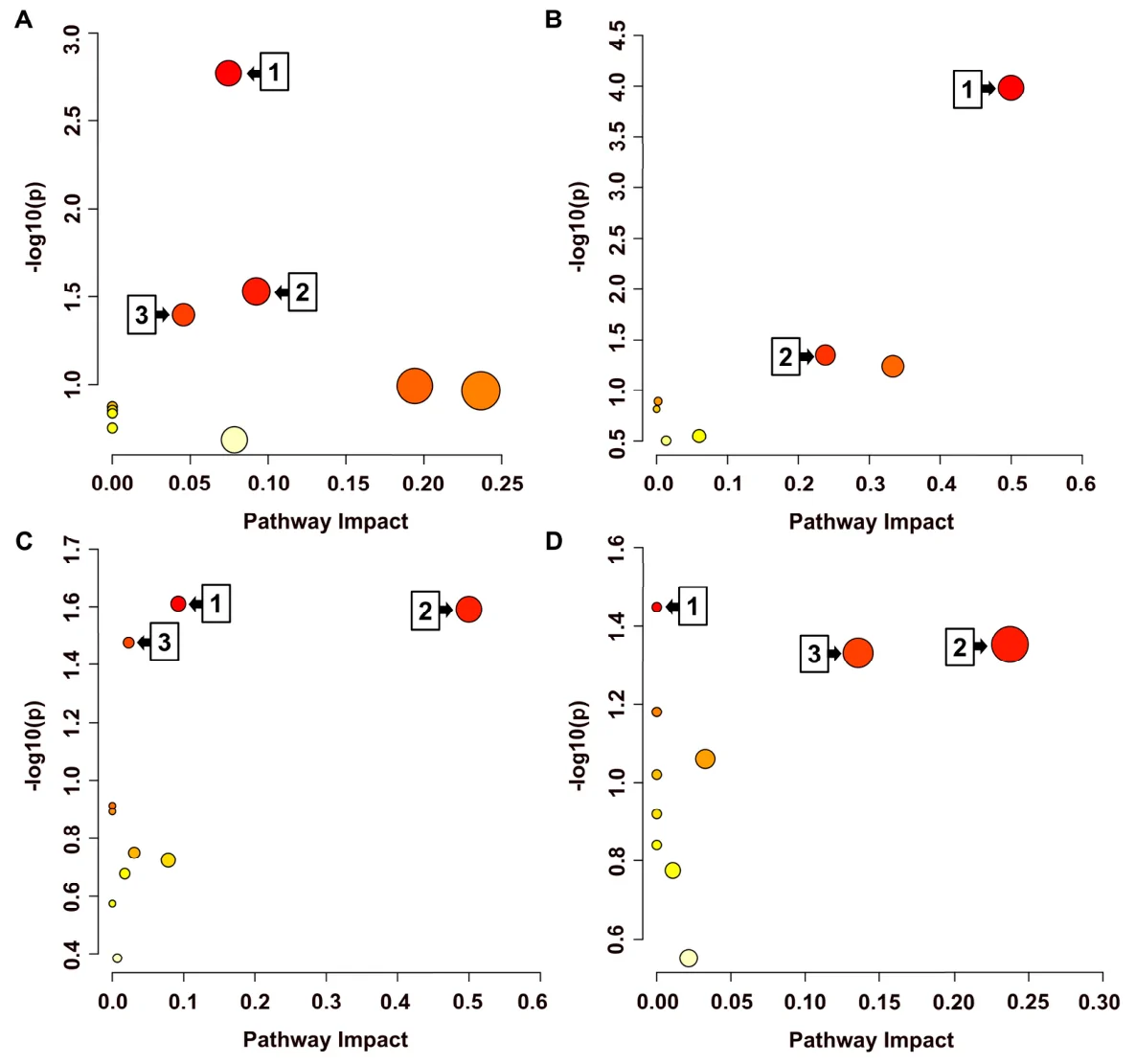
Pathway impact plot from the enrichment analysis of metabolites in the liver of rats fed different diets. Legend: (**A**) Metabolites present at higher abundance in the livers of rats fed a 35% *T. molitor* diet (Group AW) compared with rats fed a chicken hydrolysate diet (Group BW). 1—glycerophospholipid metabolism; 2—pyrimidine metabolism; 3—primary bile acid biosynthesis; (**B**) metabolites present at higher abundance in the livers of rats fed a chicken hydrolysate diet (Group BW) compared with rats fed a 35% *T. molitor* diet (Group AW): 1—riboflavin metabolism; 2—phenylalanine metabolism; (**C**) metabolites present at higher abundance in the livers of rats fed a 35% *T. molitor* diet (Group AW) compared with rats fed a standard laboratory diet (Group CW): 1—pyrimidine metabolism; 2—riboflavin metabolism; 3—primary bile acid biosynthesis; (**D**) metabolites present at higher abundance in the livers of rats fed a standard laboratory diet (Group CW) compared with rats fed a 35% *T. molitor* diet (Group AW): 1—valine, leucine and isoleucine biosynthesis; 2—phenylalanine metabolism; 3—steroid hormone biosynthesis. The position of each bubble represents the pathway *p*-value (y-axis) and pathway impact (x-axis). Color intensity corresponds to the statistical significance of enrichment (from yellow, low significance, to red, high significance), and bubble size reflects the number of matched metabolites within each pathway. Numbers indicate statistically significant pathways (*p* < 0.05).

The pathway enrichment analysis identified several significantly altered hepatic pathways between the compared groups. Compared with rats fed the chicken hydrolysate diet (BW), rats receiving the 35% *T. molitor* diet (AW) exhibited increased abundance of metabolites in three significantly altered pathways (Figure 4A). Glycerophospholipid metabolism showed the highest statistical significance and pathway impact followed by pyrimidine metabolism and primary bile acid biosynthesis. In contrast, metabolites with lower abundance in Group AW (FC < 0.83) were mainly associated with riboflavin metabolism, which showed the highest statistical significance and pathway impact, and phenylalanine metabolism (Figure 4B).

Compared with the standard diet (CW), Group AW exhibited increased abundance of metabolites (FC > 1.2) in pyrimidine metabolism, riboflavin metabolism, and primary bile acid biosynthesis. Conversely, metabolites that were more abundant in Group CW were associated with the biosynthesis of valine, leucine, and isoleucine, phenylalanine metabolism, and steroid hormone biosynthesis (Figure 4D). Among these, branched-chain amino acid biosynthesis showed the highest statistical significance and pathway impact.

**Figure 5:**
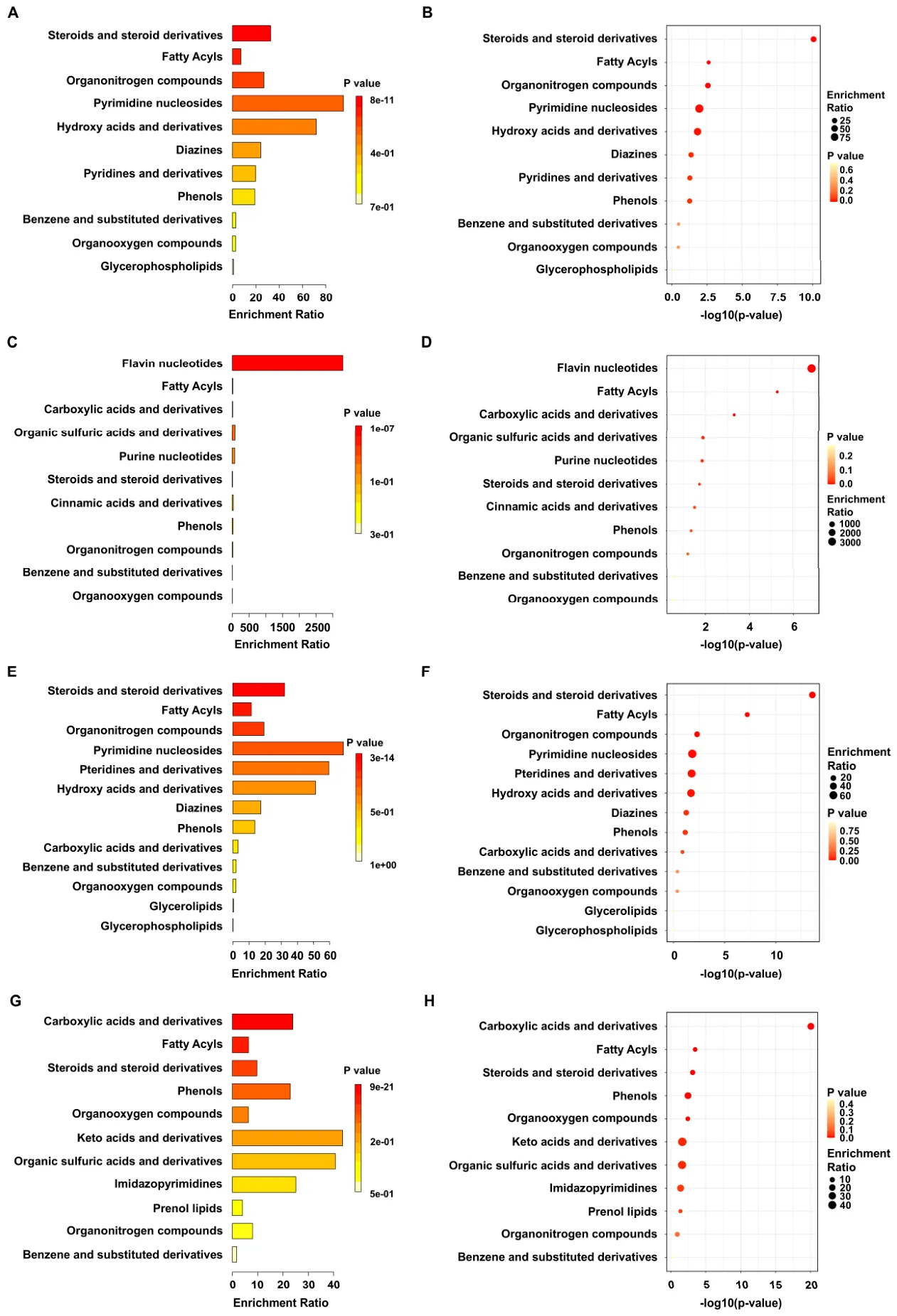
Enrichment analysis of the main chemical classes of metabolites in the livers of rats fed different diets. Legend: (**A**,**B**) Metabolites present at higher abundance in the livers of rats fed a 35% *T. molitor* diet (Group AW) compared with rats fed a chicken hydrolysate diet (Group BW); (**C**,**D**) Metabolites present at higher abundance in the livers of rats fed a chicken hydrolysate diet (Group BW) compared with rats fed a 35% *T. molitor* diet (Group AW) (**E**,**F**) metabolites present at higher abundance in the livers of rats fed a 35% *T. molitor* diet (Group AW) compared with rats fed a standard laboratory diet (Group CW); (**G**,**H**) metabolites present at higher abundance in the livers of rats fed a standard laboratory diet (Group CW) compared with rats fed a 35% *T. molitor* diet (Group AW). (**A**,**C**,**E**,**G**) Bar plots showing the enrichment ratio and corresponding *p*-values for significantly enriched metabolite classes. (**B**,**D**,**F**,**H**) Bubble plots illustrate the relationship between enrichment significance (−log_10_(*p*-value)) and enrichment ratio. The color gradient represents the *p*-value (from yellow, low significance, to red, high significance), and bubble size corresponds to the enrichment ratio.

The chemical class enrichment analysis (Figure 5A,B) demonstrated that metabolites present at higher abundance in Group AW were predominantly associated with lipid- and nitrogen-containing compound classes. The strongest enrichment was observed for pyrimidine nucleosides, followed by hydroxy acids and derivatives, organonitrogen compounds, and steroids and steroid derivatives, fatty acyls, diazines, pyridines and derivatives, and phenols were also enriched whereas glycerophospholipids, organooxygen compounds, and benzene and substituted derivatives showed relatively low enrichment.

Analysis of metabolites with lower abundance in Group AW (Figure 5C,D) revealed the strongest enrichment for flavin nucleotides, followed by carboxylic acids and derivatives, organic sulfuric acids and derivatives, and purine nucleotides. Steroids and steroid derivatives, cinnamic acids and derivatives, phenols, and organonitrogen compounds were also less abundant, although with weaker enrichment.

A similar pattern was observed in the comparison with the standard diet (Figure 5E,F), where metabolites more abundant in Group AW were mainly represented by pyrimidine nucleosides, pteridines and derivatives, hydroxy acids and derivatives, and organonitrogen compounds. Steroids, steroid derivatives, and fatty acyls were also enriched, whereas glycerophospholipids, organooxygen compounds, and benzene and substituted derivatives showed weak or no significant enrichment.

Conversely, metabolites more abundant in Group CW (Figure 5G,H) were primarily associated with carboxylic acids and derivatives, keto acids and derivatives, and organic sulfuric acids and derivatives. Additional enriched classes included phenols, steroids, steroid derivatives, fatty acyls, imidazopyrimidines, organonitrogen compounds, and prenol lipids, whereas benzene and substituted derivatives showed relatively low enrichment.

### 2.4. Spleen Metabolomics

**Figure 6:**
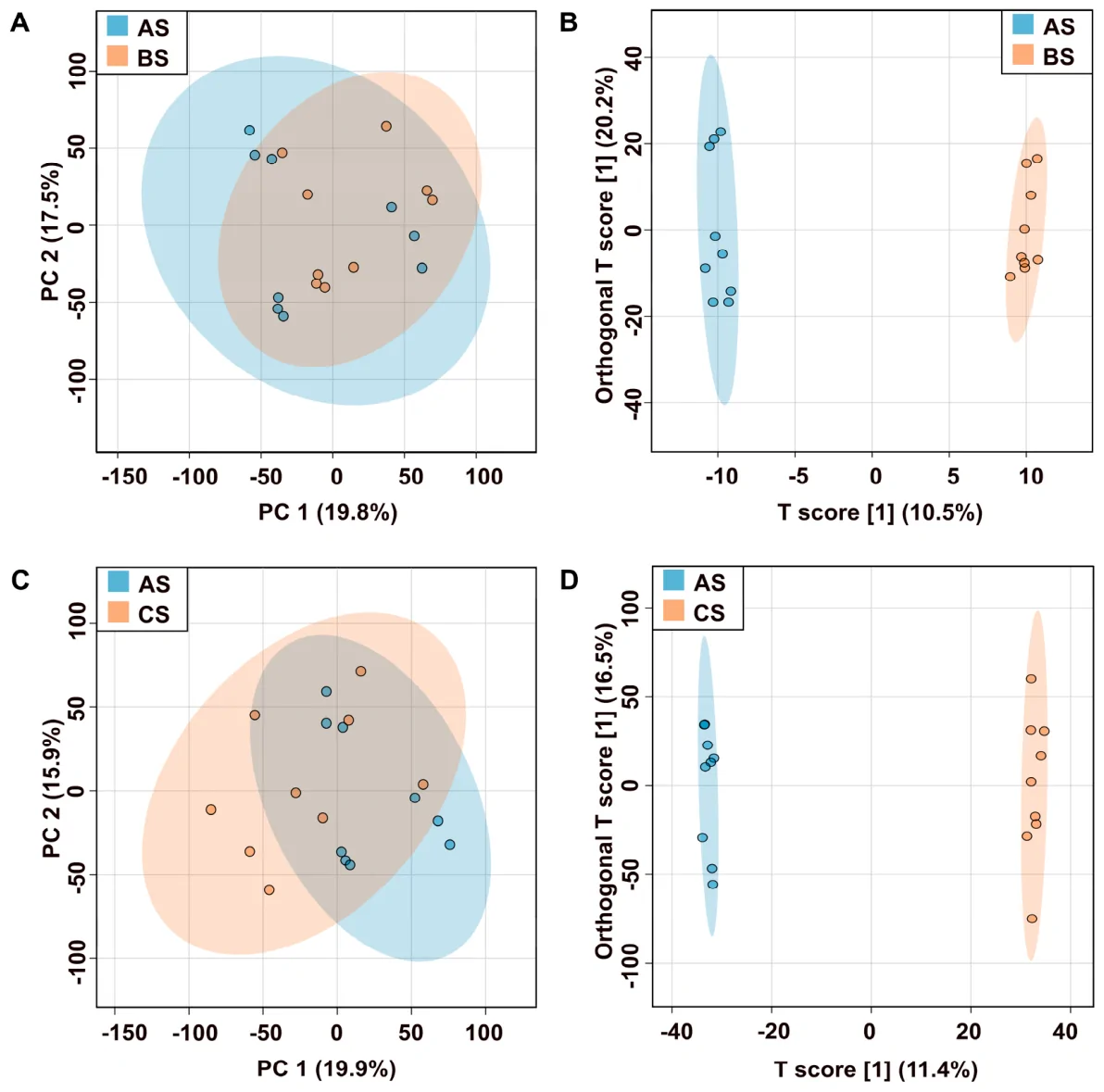
Multivariate statistical analyses of spleen metabolite profiles from rats fed a diet containing 35% *T. molitor* meal (AS) compared with rats fed control diets: a diet containing chicken hydrolysate (Group BS; panels **A** and **B**) or a standard laboratory diet (Group CS; panels **C** and **D**). Legend: Each point represents an individual sample, and the 95% confidence ellipses illustrate within-group variability. (**A**,**C**) Principal component analysis (PCA) score plots. (**B**,**D**) Orthogonal partial least squares discriminant analysis (OPLS-DA) score plots showing separation between the dietary groups.

Unsupervised PCA of the entire UHPLC-HRMS metabolomics dataset revealed differences in spleen metabolic profiles between rat fed the 35% *T. molitor* diet (AS) and the chicken hydrolysate diet (BS) (PC1: 19.8%; PC2: 17.5%), as well as partial separation between the AS and standard diet (CS) groups (PC1: 19.9%; PC2: 15.9%) (Figure 6A,C). Although partial overlap of the 95% confidence intervals was observed, the overall sample distribution indicated diet-related changes in spleen metabolite composition.

Supervised OPLS-DA confirmed clear separation between AS and BS (predictive component: 10.5%; orthogonal component: 20.2%) and between AS and CS (11.4% and 16.5%, respectively) (Figure 6B,D) confirming distinct spleen metabolite profiles among the dietary groups.

**Figure 7:**
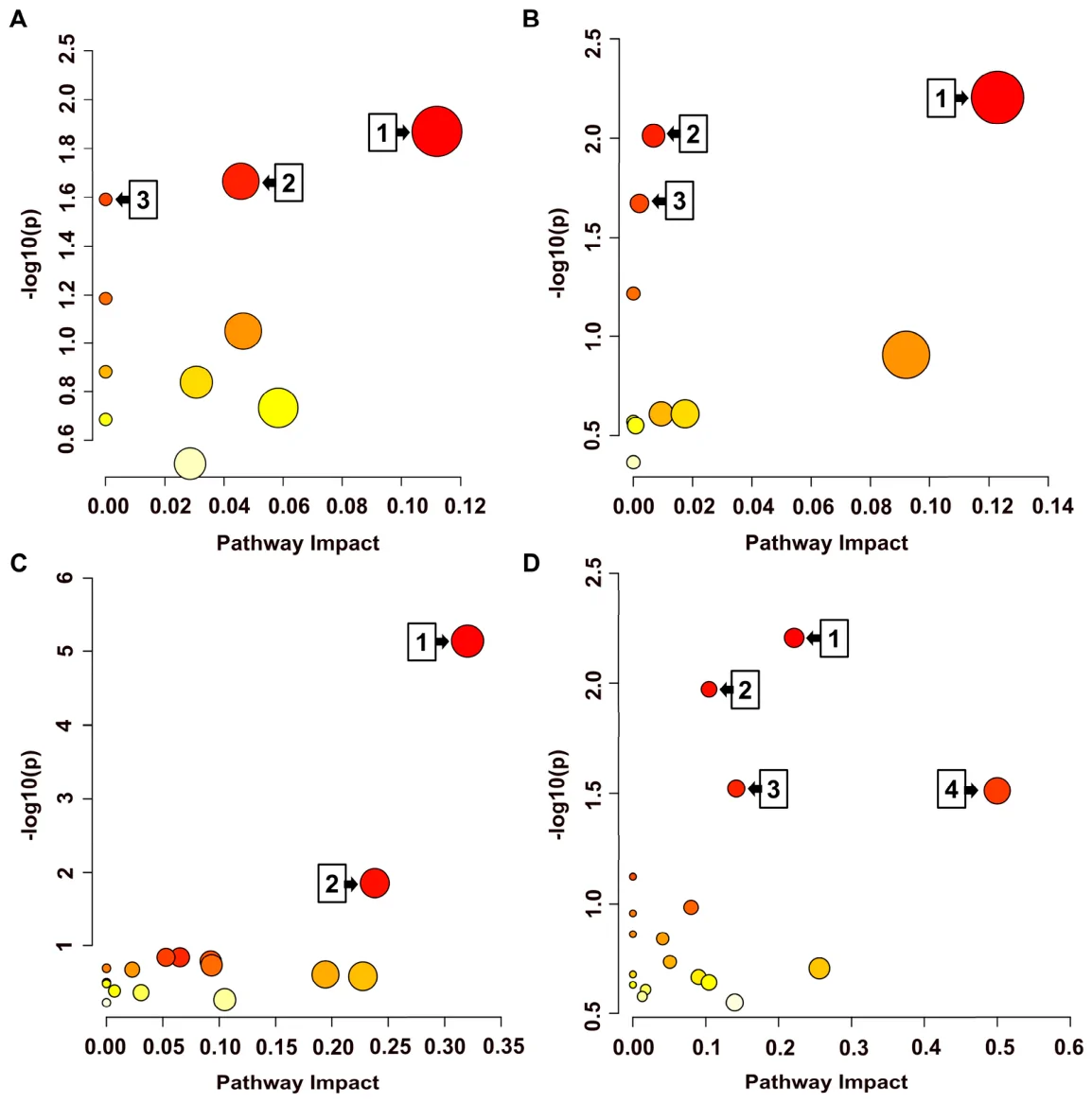
Pathway impact plots from the enrichment analysis of metabolites in the spleens of rats fed different diets. Legend: (**A**) Metabolites present at higher abundance in the spleens of rats fed a 35% *T. molitor* diet (Group AS) compared with rats fed a chicken hydrolysate diet (Group BS). 1—glycerophospholipid metabolism; 2—primary bile acid biosynthesis; 3—linoleic acid metabolism; (**B**) metabolites present at higher abundance in the spleen of rats fed a chicken hydrolysate diet (Group BS) compared with rats fed a 35% *T. molitor* diet (Group AS): 1—histidine metabolism; 2—pantothenate and CoA biosynthesis; 3—lysine degradation.; (**C**) metabolites present at higher abundance in the spleen of rats fed a 35% *T. molitor* diet (Group AS) compared with rats fed a standard laboratory diet (Group CS): 1—steroid hormone biosynthesis; 2—phenylalanine metabolism; (**D**) metabolites present at higher abundance in the spleens of rats fed a standard laboratory diet (Group CS) compared with rats fed a 35% *T. molitor* diet (Group AS): 1—histidine metabolism; 2—β-alanine metabolism; 3—arginine and proline metabolism; 4—phenylalanine, tyrosine and tryptophan biosynthesis. The position of each bubble represents the pathway *p*-value (y-axis) and pathway impact (x-axis). Color intensity corresponds to the statistical significance of enrichment (from yellow, low significance, to red, high significance), and bubble size reflects the number of matched metabolites within each pathway. Numbers indicate statistically significant pathways (*p* < 0.05).

Pathway enrichment analysis identified several significantly altered metabolic pathways. Compared with Group BS, Group AS showed higher metabolite abundance (FC > 1.2) in glycerophospholipid metabolism, primary bile acid biosynthesis, and linoleic acid metabolism (Figure 7A). In contrast, metabolites more abundant in Group BS (FC < 0.83) were mainly associated with histidine metabolism, pantothenate and CoA biosynthesis, and lysine degradation (Figure 7B), with histidine metabolism showing the highest statistical significance.

Compared with the standard diet (CS), Group AS showed increased metabolite abundance, in steroid hormone biosynthesis and phenylalanine metabolism (FC > 1.2) (Figure 7C) whereas metabolites more abundant in Group CS (FC < 0.83) were associated with histidine, β-alanine, arginine, and proline metabolism, as well as phenylalanine, tyrosine, and tryptophan biosynthesis (Figure 7D). Histidine metabolism showed the highest statistical significance, whereas phenylalanine, tyrosine, and tryptophan biosynthesis exhibited the highest pathway impact.

**Figure 8:**
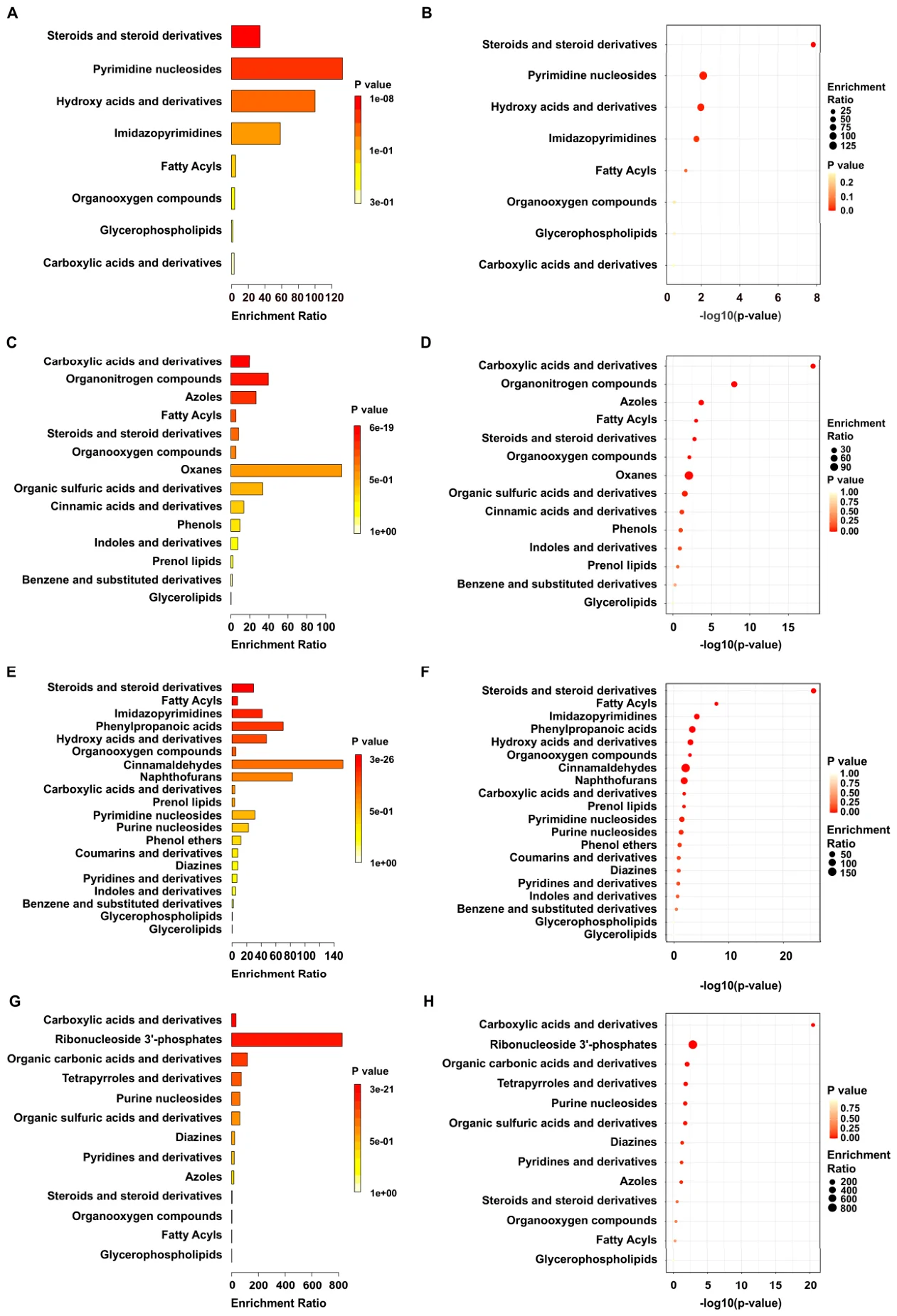
Enrichment analysis of the main chemical classes of metabolites in the spleens of rats fed different diets. Legend: (**A**,**B**) metabolites present at higher abundance in the spleens of rats fed a 35% *T. molitor* diet (Group AS) compared with rats fed a chicken hydrolysate diet (Group BS); (**C**,**D**) metabolites present at higher abundance in the spleens of rats fed a chicken hydrolysate diet (Group BS) compared with rats fed a 35% *T. molitor* diet (Group AS); (**E**,**F**) metabolites present at higher abundance in the spleen of rats fed a 35% *T. molitor* diet (Group AS) compared with rats fed a standard laboratory diet (Group CS); (**G**,**H**) metabolites present at higher abundance in the spleen of rats fed a standard laboratory diet (Group CS) compared with rats fed a 35% *T. molitor* diet (Group AS). (**A**,**C**,**E**,**G**) Bar plot showing enrichment ratios and corresponding *p*-values for significantly enriched metabolite classes. (**B**,**D**,**F**,**H**) Bubble plots illustrating the relationship between enrichment significance (−log_10_(*p*-value)) and enrichment ratio. The color gradient represents the *p*-value (from yellow, low significance, to red, high significance), and bubble size corresponds to the enrichment ratio.

The chemical class enrichment analysis (Figure 8A,B) demonstrated that metabolites present in Group AS were primarily associated with lipid- and nitrogen-containing compound particularly pyrimidine nucleosides, hydroxy acids and derivatives, imidazopyrimidines, and steroids and steroid derivatives. In contrast, fatty acyls, organooxygen compounds, glycerophospholipids, and carboxylic acids and derivatives showed relatively low enrichment.

Metabolites more abundant in Group BS (Figure 8C,D) were primarily represented by carboxylic acids and derivatives, organonitrogen compounds, azoles, oxanes, organic sulfuric acids and derivatives, and cinnamic acids and derivatives. Steroids and steroid derivatives, fatty acyls, phenols, and indoles and derivatives showed moderate enrichment, whereas prenol lipids, benzene and substituted derivatives, and glycerolipids exhibited relatively low enrichment.

Compared with the standard diet (Figure 8E,F), metabolites enriched in Group AS were mainly associated with cinnamaldehydes, naphthofurans, phenylpropanoic acids, hydroxy acids and derivatives, imidazopyrimidines, steroids and steroid derivatives, fatty acyls, organooxygen compounds, and pyrimidine nucleosides. Conversely, metabolites more abundant in Group CS (Figure 8G,H) were primarily associated with ribonucleoside 3′-phosphates, organic carbonic acids and derivatives, purine nucleosides, tetrapyrroles and derivatives, and organic sulfuric acids and derivatives, whereas diazines, pyridines and derivatives, and azoles showed moderate enrichment.

Detailed descriptive statistics, metabolite identification data, and the complete results of pathway and chemical-class enrichment analyses are provided in the Supplementary Materials (File S1; Tables S2–S22).

## 3. Discussion

### 3.1. Fecal Enzyme Analysis, Short-Chain Fatty Acid Analysis, and Assessment of Fecal Water Genotoxicity

It is important to acknowledge that while our findings demonstrate significant diet-induced changes in the functional activity of the gut environment, they do not provide direct evidence of altered gut microbial composition. The inclusion of 35% *T. molitor* markedly altered colonic fermentation compared with the chicken hydrolysate and standard diets. Insect-fed rats showed significantly lower concentrations of formate, acetate, butyrate, valerate, and BCFAs, together with higher β-glucosidase and β-glucuronidase activity. These findings indicate that the complete *T. molitor*-based dietary formulation modified the functional activity of the gut environment compared with the chicken hydrolysate and standard diets.

Unlike conventional rodent diets, in which carbohydrates are primarily derived from purified starches or cereal grains, *T. molitor* meal provides a substantial chitin fraction in addition to protein and fat [44,45]. The composition of *T. molitor* meals varies depending on processing, particularly with respect to protein, fat, and chitin content [46,47,48]. Chitin may reduce nutrient digestibility by encapsulating proteins and increasing digesta viscosity, thereby allowing more protein to escape digestion in the small intestine [49]. Unlike most plant fibers, insect-derived chitin appears to be less readily fermented by the mammalian gut microbiota and has shown inconsistent effects on SCFA production [50,51]. For example, chitin-glucan supplementation increased cecal SCFA concentrations in humans [52], whereas the *T. molitor* diet reduced fecal SCFA levels in the present study. Similarly, cricket powder consumption has been associated with lower fecal acetate and propionate concentrations [53]. Together, these findings suggest that insect-derived fiber may provide a less fermentable substrate and alter microbial fermentation, resulting in lower SCFA production.

Our previous metagenomic analysis of rats fed a *T. molitor* diet demonstrated marked shifts in gut microbiota composition, including an enrichment of *Firmicutes* spp. and a reduction in *Bacteroidetes* spp. and *Akkermansia* spp. [54]. Several of the enriched taxa are involved in protein, cholesterol, and bile acid metabolism, whereas members of the Ruminococcaceae family have been associated with acetate production [55]. These microbiota changes may explain the higher β-glucosidase activity observed in the present study. Similarly, cricket powder supplementation has been shown to increase *Bifidobacterium animalis*, a species exhibiting β-glucosidase activity, while reducing *Lactobacillus* spp. [53]. Although bacterial composition was not assessed in the present study, the increased β-glucosidase activity suggests altered microbial carbohydrate metabolism in response to the insect-based diet. β-Glucuronidase activity was also consistently higher in the *T. molitor* group and decreased over time. However, the absence of a significant group vs. time interaction indicates that the time pattern of change was broadly comparable among dietary treatments. These findings suggest a diet-associated difference in microbial glucuronide-hydrolyzing activity, although its biological implications cannot be determined from enzyme activity alone.

Overall, the *T. molitor* diet reduced fecal SCFA and BCFA concentrations, indicating lower fermentative activity than the chicken hydrolysate diet. In contrast to previous studies in poultry and pigs, where low dietary inclusion of insect meal increased SCFA production, particularly butyrate [56,57], the present study showed reduced SCFA concentrations. This finding also contrasts with the increase in BCFAs typically associated with enhanced proteolytic fermentation [58,59] and may reflect differences in substrate availability or microbial metabolism. The marked reduction in butyrate concentrations observed in the insect-fed group is of particular physiological relevance. Butyrate is the primary energy source for colonocytes and plays a critical role in maintaining intestinal barrier integrity, modulating immune responses, and exerting anti-inflammatory effects [60,61,62]. Therefore, the lower butyrate levels in Group A should be interpreted as a negative outcome in isolation. While the insect-based diet was associated with reduced fecal water genotoxicity, this positive finding does not negate the potential disadvantages of diminished butyrate production. The net effect on colonic health likely depends on the balance between reduced fecal water genotoxicity and diminished butyrate production and warrants further investigation, including assessment of intestinal barrier function and inflammatory markers.

Rats fed the *T. molitor* diet showed a marked reduction in fecal water genotoxicity at week 6, suggesting a lower genotoxic burden in the intestinal environment. By the end of the experiment, genotoxicity levels were comparable between the insect-fed and standard diet groups, whereas the chicken hydrolysate diet remained associated with the highest genotoxicity. Multivariable regression analysis supported a time-dependent association between the overall microbial metabolic profile and fecal water genotoxicity, although the contribution of individual SCFAs could not be distinguished because of multicollinearity.

By the end of the experiment, fecal water genotoxicity was comparable in the *T. molitor* and standard diet groups, whereas the chicken hydrolysate diet remained associated with significantly higher genotoxicity. These findings suggest that diets containing fermentable substrates, including insect-derived chitin, may contribute to a less genotoxic colonic environment [63,64,65]. Dietary fiber is known to reduce fecal water genotoxicity by modulating gut microbial activity and limiting exposure to potentially harmful metabolites [64,65]. Although the underlying mechanisms were not investigated in the present study, the persistent elevation of genotoxicity between the studied groups may therefore reflect differences in the complete dietary matrices.

The differences observed between the *T. molitor* and chicken hydrolysate diets may reflect distinct patterns of gut microbial metabolism [66]. Diet is a major determinant of gut microbiota composition and metabolic activity, and high-protein diets with limited fermentable substrates have been associated with the production of potentially harmful microbial metabolites [66,67]. Consistent with this, the chicken hydrolysate diet remained associated with higher fecal water genotoxicity throughout the study. In addition, insect-derived compounds have been reported to modulate gut microbial composition [42], which may have contributed to the lower genotoxicity observed in the *T. molitor* group. However, the underlying mechanisms were not investigated in the present study.

### 3.2. Metabolomics

#### 3.2.1. Liver

One of the most prominent metabolic changes observed in the *T. molitor* group was the enrichment of pathways related to lipid metabolism, particularly glycerophospholipid metabolism and primary bile acid biosynthesis. Rats fed the insect-based diet showed higher levels of glycerophosphocholine, choline phosphate, and related glycerophospholipid metabolites, indicating differences in the hepatic metabolite profile associated with phospholipid metabolism. These changes are consistent with the high lipid content of *T. molitor* meal [43] and may potentially reflect differences in hepatic handling of dietary lipids. However, their physiological significance cannot be determined based on the available metabolomic data.

The *T. molitor* diet was associated with enrichment of metabolites assigned to primary bile acid biosynthesis, including higher levels of glycocholate and glycochenodeoxycholate. These changes may potentially reflect the fatty acid profile of the insect-based diet and are consistent with previous reports showing hypolipidemic effects of *T. molitor*, chitosan, and chitin in animal models [68,69,70]. Overall, the functional consequences of these metabolic changes could not be determined in the present study, as blood lipid concentrations and bile acid metabolism were not directly assessed.

Enrichment of metabolites assigned to steroid hormone biosynthesis represented another feature of the hepatic metabolic profile in the *T. molitor* group. Rats fed the standard diet exhibited higher levels of steroid precursors, including pregnenolone and 17α-hydroxyprogesterone, than insect-fed rats. However, because neither steroid hormone concentrations nor cholesterol metabolism were directly assessed, the physiological significance of these changes remains unclear and requires further investigation [71,72].

The metabolic effects of insect-based diets appear to depend on both the experimental model and the level of dietary inclusion. In obese rodents, *T. molitor* supplementation has been associated with reduced hepatic lipid accumulation, inhibition of lipogenesis, and changes in phospholipid metabolism [68,73,74,75]. In contrast, studies in healthy pigs reported only minor alterations in liver lipid composition and no significant changes in plasma bile acid levels following dietary inclusion of mealworm meal [74]. In broiler chickens, however, *T. molitor* supplementation altered bile acid metabolism [76], supporting our observation of enriched bile acid-related pathways in the liver. Collectively, these findings suggest that the metabolic response to insect-based diets is influenced by species, physiological status, and dietary inclusion level. Nevertheless, the mechanisms underlying these differences remain unclear and may also involve diet-induced changes in gut environment [55].

Beyond lipid metabolism, the *T. molitor* diet was also associated with significant enrichment of metabolites assigned to pyrimidine metabolism. Higher hepatic levels of uridine and uracil, together with lower hypoxanthine concentrations, indicate differences in the hepatic nucleotide-related metabolite profile compared with the control diets [77,78,79]. A possible explanation is the higher intake of nucleic acid-derived compounds from whole *T. molitor* meal, which contains nucleic acids absent from purified protein hydrolysates [80,81,82,83]. Changes in gut microbial metabolism may also have contributed to this metabolic profile [84,85]. However, because neither nucleotide absorption nor microbial nucleotide metabolism was assessed, the underlying mechanisms and physiological significance of these findings remain uncertain. Although uridine has been associated with beneficial metabolic effects in previous studies [86], such functional implications cannot be inferred from the present data.

Significant differences in metabolites assigned to amino acid-related pathways were also observed between dietary groups. Phenylalanine metabolism was less represented in the *T. molitor* group than in the chicken hydrolysate and standard diet groups. One possible explanation is that amino acids derived from whole insect meal become available more gradually than those released from hydrolyzed chicken protein, resulting in altered phenylalanine metabolism [16,87,88]. This interpretation is consistent with the moderate reduction in protein digestibility associated with chitin-containing diets [89,90]. In addition, the biosynthesis pathway of valine, leucine, and isoleucine was relatively enriched in rats fed the standard diet, which also exhibited higher levels of 3-methyl-2-oxopentanoate and a tendency toward increased succinate concentrations. Together, these findings indicate that the insect-based diet was associated with an altered hepatic amino acid-related metabolite profile, potentially reflecting differences in protein composition and digestibility compared with conventional components [47,91,92]. Nevertheless, the mechanisms underlying these metabolic changes were not investigated in the present study and therefore remain speculative.

Metabolites associated with vitamin cofactor pathways also differed between the insect- and chicken hydrolysate diets. Group A showed lower hepatic levels of flavin adenine dinucleotide (FAD) and flavin mononucleotide (FMN), and riboflavin metabolism was identified as one of the discriminating pathways. Although *T. molitor* is considered a good source of riboflavin [93,94], the observed differences may reflect variations in nutrient bioavailability, diet composition, or metabolic utilization. However, because dietary riboflavin content, vitamin status, and flavin-dependent enzyme activity were not assessed, the biological significance of these findings remains uncertain. Jiang et al. [76] reported increased thymine levels in the cecal contents of broilers fed *T. molitor*, suggesting that insect-derived ingredients may influence nucleotide metabolism. Further studies are needed to determine whether these metabolomic differences reflect functional changes in vitamin metabolism [58,93,94,95,96].

Overall, the *T. molitor* diet was associated with differences in hepatic metabolites assigned to lipid-, amino acid-, and nucleotide-related pathways compared with the control diets. The enrichment of lipid-related metabolites is consistent with previous studies reporting effects of insect-based diets on hepatic lipid homeostasis, including reduced lipogenesis and hepatic steatosis [68,73,97]. At present, the functional significance of these alterations cannot be determined from untargeted metabolomic data alone and requires confirmation by targeted biochemical and physiological analyses [98,99].

The enrichment of glycine-conjugated bile acids is consistent with normal hepatic bile acid metabolism rather than the accumulation of highly cytotoxic unconjugated bile acids [100,101,102]. Likewise, the observed alterations in amino acid metabolism are consistent with previous reports demonstrating that insect-based diets modify amino acid utilization without markedly disrupting amino acid homeostasis [74,75]. However, because the present study was based on untargeted metabolomic profiling, the functional significance of these alterations cannot be determined and requires confirmation in future studies [103,104].

#### 3.2.2. Spleen

The spleens of rats fed the *T. molitor* diet exhibited distinct metabolomic differences, particularly among metabolites associated with lipid-related pathways. Pathway analysis identified enrichment of metabolites assigned to glycerophospholipid and fatty acid metabolism compared with the control groups. Enrichment of metabolites assigned to linoleic acid metabolism may be relevant to lipid mediator pathways. However, the present data do not demonstrate functional changes in these processes or in splenic immune activity [105].

Sterol-, steroid-, and bile acid-related pathways were also enriched in the spleens of rats fed the *T. molitor* diet. Bile acids are important metabolic signaling molecules involved in immune regulation [106,107]. Because the spleen is not a primary site of bile acid synthesis, these findings may potentially reflect systemic exposure to bile acid-related metabolites or metabolic signaling, and not de novo bile acid production. Similarly, enrichment of steroid-related metabolites indicates differences in the abundance of compounds assigned to steroid metabolism. However, it does not demonstrate functional activation of steroid metabolism or splenic immune responses [108].

The abundance of metabolites assigned to nucleotide-related pathways also differed between dietary groups. Rats fed the *T. molitor* diet exhibited enrichment of metabolites assigned to pyrimidine metabolism, whereas the standard diet was associated with higher levels of purine-related metabolites. Because nucleotide turnover, cell proliferation, and immune cell activity were not directly assessed, the biological significance of these metabolic differences remains uncertain and requires further investigation. Differences in metabolites assigned to amino acid-related pathways were also evident in the spleen. Metabolites assigned to histidine metabolism were less represented in rats fed the *T. molitor* diet than in either control group, indicating a different amino acid-related metabolite profile in response to the insect-based diet. Lower dietary histidine content in *T. molitor* meal may partly explain these differences [109,110]. In addition, pathways related to the metabolism of aromatic amino acids, including phenylalanine, tyrosine, and tryptophan, as well as lysine degradation and pantothenate/CoA biosynthesis, were less represented in the insect-fed group. However, the physiological and immunological significance of these changes cannot be determined from metabolomic profiling alone and requires further investigation.

To the best of our knowledge, this is the first study to characterize the splenic metabolome in animals fed an insect-based diet. Previous studies evaluating *T. molitor* or *H. illucens* have primarily focused on growth performance, nutrient digestibility, immune parameters, or metabolomic changes in plasma, liver, and muscle rather than splenic tissue [74]. Meyer et al. [74] reported only modest metabolic changes in pigs fed diets containing 5–10% *T. molitor*. In contrast, the present study demonstrated distinct differences in the splenic metabolome, particularly among metabolites associated with lipid-, nucleotide-, and amino acid-related pathways, following dietary inclusion of 35% *T. molitor* meal. These differences may potentially reflect the substantially higher dietary inclusion level used in the present study and highlight the need for further investigations into tissue-specific metabolic responses to insect-based diets.

### 3.3. Integrated Assessment of Gut Environment Functional Activity and Metabolic Profiles

Rats fed the 35% *T. molitor* diet exhibited a distinct fermentation profile and lower fecal water genotoxicity than rats fed the chicken hydrolysate diet. Although concentrations of several SCFAs, including butyrate, were reduced, the simultaneous decrease in fecal water genotoxicity suggests that the altered fermentation profile was not accompanied by an increased genotoxic burden in the intestinal environment. Nevertheless, the lower butyrate concentration should be interpreted cautiously because of its importance for colonic epithelial homeostasis. Therefore, the overall implications of the insect-based diet for gut health require further investigation.

These gut-derived changes coincided with diet-dependent differences in liver and spleen metabolite profiles. In the liver, the main differences involved metabolites assigned to lipid-, bile acid-, nucleotide-, amino acid-, and vitamin cofactor-related pathways, whereas splenic differences primarily involved lipid-, steroid-, nucleotide-, and amino acid-related metabolites. These concurrent diet-associated changes may be considered in the context of a potential gut–liver–spleen axis, in which diet-related changes in the intestinal environment may be associated with metabolic responses in the liver and spleen. However, the present study does not establish causal communication between these organs, and pathway enrichment should not be interpreted as evidence of functional activation of the corresponding hepatic or splenic processes. Overall, the findings indicate concurrent diet-associated differences in gut functional markers and peripheral organ metabolite profiles rather than isolated responses within individual tissues. Their physiological and immunological significance requires confirmation using targeted metabolomics together with biochemical, functional, and immune-related measurements.

### 3.4. Limitations

A significant limitation of this study is the inability to separate the effect of the protein source from the effect of the food matrix. Although both experimental diets were formulated to achieve comparable levels of nutritional equivalence, it does not imply equivalence in terms of digestibility, peptide profile, amino acid absorption kinetics, fiber composition, or fermentability. The full-fat, non-dechitinized *T. molitor* meal provided intact insect proteins, chitin, insect lipids, and other larval components, whereas the reference diet contained chicken hydrolysate alongside separately balanced lipid and fiber sources. Therefore, the observed intestinal and systemic responses should be interpreted as the effects of the complete dietary formulations rather than effects attributable solely to insect protein versus chicken hydrolysate. Further factorial studies using isolated insect protein, defatted or dechitinized insect meals, purified chitin, and matched animal protein preparations are required to determine the contribution of each dietary component. The dietary design should also be considered in the context of the translational and animal-welfare objectives of the project. The primary requirement of the feeding trial was to provide nutritionally balanced complete diets that could be administered safely throughout the experimental period. The present rat experiment represented a preclinical evaluation of the complete feeds rather than a comparison of purified dietary fractions. Such diets have to meet specific nutritional values. Removing chitin, isolating the insect protein, or independently modifying the lipid and carbohydrate components would have altered the nutritional balance and produced formulations that no longer represented the intended clinical product. Furthermore, distinguishing the individual effects of protein origin, hydrolysis, chitin, and lipid composition would require a separate factorial study with additional experimental groups and animals.

Another limitation is that only a single inclusion level of *T. molitor* meal was evaluated. Consequently, this study does not allow for the determination of a dose–response relationship or for ascertaining whether comparable intestinal or systemic responses would occur at lower inclusion levels. Furthermore, the metabolomic analysis was conducted on a limited number of biological samples, with three samples per organ analyzed in each dietary group. Although each sample was analyzed in triplicate, the small number of biological samples limits the statistical power and the generalizability of the metabolomic analysis results.

An additional limitation is the lack of assessment regarding direct indicators of inflammation, oxidative stress, and intestinal barrier integrity. Although metabolomic analysis revealed differences in diet-related metabolites, associated with pathways involving lipids, bile acids, nucleotides, and amino acids, these results do not determine whether the observed profiles reflect a beneficial physiological adaptation, metabolic stress, immune system modulation, or another non-specific response to the dietary regimens. The functional interpretation of the findings would be enhanced by measurements of circulating and tissue-based inflammatory mediators, markers of oxidative stress and antioxidant defense, as well as assessments of intestinal permeability, tight junction proteins, and intestinal histomorphology. Consequently, the physiological and immunological significance of the observed metabolic differences remains unclear and requires confirmation through studies combining metabolomics with targeted biochemical, immunological, and histological analyses.

## 4. Materials and Methods

### 4.1. Feed Preparation

Complete feed was formulated in accordance with the nutritional recommendations of the European Pet Food Industry Federation (FEDIAF). Live *Tenebrio molitor* larvae were obtained from a domestic insect producer (Tenebria, Lubawa, Poland) operating under a Hazard Analysis and Critical Control Points (HACCP) program. The facility complies with applicable Polish veterinary legislation governing the safety of food of animal origin (Official Journal of Laws of the Republic of Poland 2019, item 1252), animal health and infectious disease prevention and control (Official Journal of Laws of the Republic of Poland 2020, items 148 and 285), animal welfare (Official Journal of Laws of the Republic of Poland 2019, items 122 and 1123), and feed (Official Journal of Laws of the Republic of Poland 2019, item 269).

Before processing, larvae were fasted for 72 h to empty the gastrointestinal tract. They were subsequently mechanically cleaned to remove residual substrate, frass, shed cuticles, and any dead or pupated specimens. Larvae were euthanized using thermal procedures consistent with the American Veterinary Medical Association (AVMA) Guidelines for the Euthanasia of Animals. The resulting meal was produced on a dedicated processing line designed for insect stunning/killing and full processing. Feed was produced under a Quality Management System aligned with ISO 9001 requirements and HACCP principles. The formulated diets consisted of dry oval pellets with a diameter of approximately 15 mm.

Two experimental diets were prepared: (i) an insect-based diet containing 35% full-fat, non-dechitinized *T. molitor* meal, and (ii) a chicken hydrolysate diet containing commercially available chicken hydrolysate and fish oil. The nutritional composition of these diets was designed to meet the following target parameters on a dry matter basis: crude protein content of approximately 28% (±2.5%); crude fat content of approximately 15% (±1.5%); crude ash content of approximately 5% (±2.5%), and crude fiber content of approximately 5% (±2.5%). The 35% *T. molitor* meal inclusion level was selected prospectively as the target concentration for the complete hypoallergenic formulation developed within the project. This level enabled *T. molitor* meal to serve as the principal animal-derived ingredient while allowing the diet to meet its predefined nutritional targets and remain suitable for industrial dry-feed extrusion. Previous formulation studies evaluating 25–45% inclusion demonstrated that 35% provided a practical balance between substantial insect-meal incorporation, nutritional composition, and granule properties, whereas further increases progressively altered nutrient balance and produced softer, more deformable granules. The suitability of this inclusion range was additionally supported by a preceding graded-dose rat feeding trial. Accordingly, 35% was selected as the technologically, nutritionally, and translationally justified inclusion level for subsequent preclinical evaluation [91,111]. Therefore, this level was selected as a practical formulation-specific concentration for the present translational study. The experimental diets for Groups A and B were formulated to be approximately isoenergetic and isonitrogenous at the proximate level. All of the remaining ingredients were similar in both formulations. The complete recipe is the subject of Patent no. 247885. The diets were designed as hypoallergenic options for dogs with food-responsive enteropathies [91] and this animal trial was conducted as a preclinical evaluation with multiple endpoints. Detailed data on the nutritional composition of the feeds used in the experiment, and other relevant information can be found in the Supplementary Materials (Table S23).

### 4.2. Laboratory Animals and Experimental Procedure

The experiment was designed and performed in line with the 3R framework (Replacement, Reduction, and Refinement) [112,113]. Reporting followed ARRIVE (Animal Research: Reporting of In Vivo Experiments) recommendations. Ethical approval was granted by the Local Ethics Committee (decision No. 31/2022 of 18 May 2022), and all procedures complied with national regulations (Act of 15 January 2015 on the protection of animals used for scientific or educational purposes; Official Journal of 2015, item 266) as well as Directive 2010/63/EU of 22 September 2010 on the protection of animals used for scientific purposes.

Wistar rats (*Rattus norvegicus*) served as the in vivo model because they are widely used in physiological and nutritional studies relevant to gastrointestinal research. The study included 12-month-old female rats (250–280 g) obtained from a single breeding source (*n* = 45). The animals underwent a 14-day acclimation/quarantine period prior to the start of the trial.

Throughout acclimation and the experimental phase, the rats were kept under standardized husbandry conditions: the cages had at least 800 cm^2^ of floor space and a minimum height of 18 cm, ambient temperature was maintained at 22 ± 2 °C with relative humidity of 60 ± 5%, and lighting followed a 12 h light/12 h dark schedule at approximately 65 lux. The animals were housed in a dedicated laboratory animal facility meeting standard requirements. They were allocated to three groups (*n* = 15 per group) and housed individually: Group A (experimental) received a diet containing 35% *T. molitor* meal; Group B (positive control) was fed a diet containing chicken hydrolysate; and Group C (negative control) received a standard laboratory diet (Labofeed H Standard, Wytwórnia Pasz Morawski, Żurawia, Poland). Randomization was performed using a computerized random generator, which ensured unbiased assignment of animals to experimental groups. Animals were housed individually in transparent cages that enabled visual contact with other rats in order to support behavioral needs. The feeding period lasted 60 days. Daily rations were adjusted to meet the energy needs of animals, and water was available ad libitum. Detailed data on body weight progression and feed intake are presented in Supplementary Materials (Table S24). Fecal samples were collected after 4, 6, and 8 weeks of feeding. Freshly excreted fecal pellets were immediately placed in sterile tubes and subjected to further analysis. At the end of the experiment, the rats were euthanized by isoflurane overdose, and liver and spleen samples were collected. The study had a single-blind design as the animal handlers and laboratory personnel performing experimental analyses were blinded to the group allocation. Due to practical and organizational constraints of the study, the principal investigator and the person responsible for data storage were aware of the group allocation.

### 4.3. Analysis of Short-Chain Fatty Acids and Fecal Enzymes

#### 4.3.1. Determination of Fecal Enzyme Activity

One gram of each fecal sample was weighed into Falcon tubes and brought to a final volume of 10 mL with phosphate buffer (pH 7.0). The samples were thoroughly mixed and sonicated (A = 60, t = 2 min, s = 6 s). Next, they were centrifuged for 20 min at 4 °C and 14,000 rpm (MPW-380R centrifuge, MPW, Warszawa Poland). The supernatant was stored at −20 °C until analysis. The prepared samples were used to determine β-glucuronidase and β-glucosidase activity.

#### 4.3.2. Protein Determination

Protein content was determined in a 96-well microplate; 5 µL of the tested sample and 250 µL of Bradford reagent were added to each well. The samples were incubated at 27 °C for 10 min. Absorbance was then measured at λ = 540 nm using a Multiskan GO Microplate Spectrophotometer (Thermo Scientific, Waltham, MA, USA) and reagent blanks containing distilled water instead of the sample. Protein concentration was calculated from a calibration curve (y = 0.8889x) prepared using bovine serum albumin (BSA; Quick Start Bovine Serum Albumin) under the assay conditions. Measurements were performed in triplicate.

#### 4.3.3. β-Glucuronidase Activity Assay

The assay was performed in a 96-well microplate; 43.9 µL of 0.02 M phosphate buffer (pH 7.0), 4.4 µL of 1 mM EDTA, 4.4 µL of 10 mM phenolphthalein-β-D-glucuronide substrate solution, and 21.9 µL of the tested sample were added to each well. The reaction mixtures were thoroughly mixed and incubated at 37 °C for 15 min. The reaction was stopped by adding 175 µL of 0.2 M glycine buffer (pH 10.4). Absorbance was measured at λ = 540 nm using a Multiskan GO Microplate Spectrophotometer (Thermo Scientific, Waltham, MA, USA). Phenolphthalein concentration was determined from a standard curve (y = 554.34x) prepared from a dilution series under the assay conditions. β-glucuronidase activity was expressed as mmol per 1 mg of protein. Measurements were performed in triplicate.

#### 4.3.4. β-Glucosidase Activity Assay

The assay was performed in a 96-well microplate; 37.9 µL of 0.2 M phosphate buffer (pH 7.0), 3.8 µL of 20 mM *p*-nitrophenyl-β-D-glucopyranoside substrate solution, and 18.9 µL of the tested sample were added to each well. The reaction mixtures were thoroughly mixed and incubated at 37 °C for 60 min. The reaction was stopped by adding 189 µL of 0.01 M NaOH. Absorbance was measured at λ = 540 nm using a Multiskan GO Microplate Spectrophotometer (Thermo Scientific, Waltham, MA, USA). A reference sample was prepared for each sample to avoid bias caused by the color of intestinal contents. In the reference sample, the enzyme was inactivated with 0.01 M NaOH immediately after the addition of the remaining components of the reaction mixture. *p*-nitrophenol concentration was determined from a standard curve (y = 171.04x + 0.0332) prepared from a dilution series under the assay conditions. β-glucosidase activity was expressed as mmol per 1 mg of protein. Measurements were performed in triplicate.

#### 4.3.5. Short-Chain Fatty Acid Determination

One gram of each fecal sample was weighed into Falcon tubes, and 5 mL of distilled water was added. The samples were shaken for 20 min at 400 rpm (Lauda shaker, LAUDA Dr. R. Wobser GmbH & CO. KG, Lauda-Königshofen, Germany) and then centrifuged for 20 min at 4 °C and 14,000 rpm (MPW-380R centrifuge, MPW, Warszawa Poland). To deproteinize the samples, 0.7 mL of the sample and 0.1 mL of Carrez reagent I were added to Eppendorf tubes and mixed. After 2 min, 0.1 mL of Carrez reagent II and 0.5 mL of distilled water were added. After mixing, the samples were centrifuged for 5 min at 10,000 rpm (DLAB D2012 Plus centrifuge, DLAB Scientific Co., Ltd., Beijing, China). The samples were then filtered through 0.22 µm pore-size filters and stored at −20 °C until analysis. The prepared samples were used for SCFA determination.

#### 4.3.6. Short-Chain Fatty Acid Analysis

Short-chain fatty acids were analyzed using a Shimadzu liquid chromatograph equipped with a Photodiode Array UV-Vis Detector (SPD-M40, Shimadzu Corporation, Kyoto, Japan). The compounds were separated on a Repromer H column (300 × 8 mm, 9 µm, Dr. Maisch HPLC GmbH, Ammerbuch-Entringen, Germany) at 60 °C, using 0.005 M sulfuric acid as a mobile phase with a flow rate of 0.7 mL/min. The concentration of each compound (formic acid, acetic acid, propionic acid, butyric acid, isobutyric acid, valeric acid, and isovaleric acid) was determined using the external standard method.

### 4.4. Genotoxicity Assessment

#### 4.4.1. Fecal Water Preparation

Freshly obtained fecal samples were mixed with sterile phosphate-buffered saline (PBS, pH 7.2) at a ratio of 1 g to 5 mL, homogenized (20 min, 400 rpm, room temperature), and centrifuged (23,666× *g*, 10 min, 4 °C). The supernatants were filtered through 0.22 µm pore-size filters and stored at −20 °C until analysis.

#### 4.4.2. IEC-6 Cell Culture

Normal rat small intestinal epithelial (IEC-6) cells were cultured as a monolayer in T75 Roux flasks in a mixture of low-glucose Dulbecco’s Modified Eagle’s Medium (DMEM) and RPMI 1640 medium (1:1, *v*/*v*), supplemented with 10% fetal bovine serum (FBS), 2 mM Gluta-MAXTM, 25 mM HEPES, and 0.1 U/mL insulin. The cells were maintained at 37 °C in 5% CO_2_ in a humidified incubator (Galaxy 48S, New Brunswick, United Kingdom) for 7 days. Every 2 days, the cells were washed with sterile PBS (pH 7.2) without Ca and Mg, and the medium was replaced. After reaching 80% confluence, the cells were detached using TrypLETM Express (37 °C, 8–10 min) and centrifuged (307× *g*, 5 min). Cell counts were determined using a hemocytometer, and cell viability was assessed with trypan blue dye. Cell viability was approximately 90% prior to the experiment.

#### 4.4.3. Single-Cell Gel Electrophoresis Assay

The final concentration of IEC-6 cells in each sample was adjusted to 10^5^ cells/mL, and the final sample volume in each Eppendorf tube was 1 mL. Cells in the negative control were suspended only in the vehicle. The cells were exposed to 20% fecal water (*v*/*v*) at 37 °C for 60 min. The comet assay was performed under alkaline conditions (pH > 13). After exposure, the cells were centrifuged (182× *g*, 15 min, 4 °C), decanted, and suspended in 0.75% low melting point (LMP) agarose. The suspension was spotted onto slides double-precoated with 0.5% normal melting point (NMP) agarose, and covered with coverslips (37 °C, ZF6 Premiere Slide Warmer hot plate, North Rocks, Australia). The samples were then placed on a Chilling Plate for Comet Assay Slides (4 °C, Cleaver Scientific, Rugby, United Kingdom), where they solidified. Next, the slides were transferred to a cuvette and lysed at 4 °C for 60 min in lysis buffer (2.5 M NaCl, 1% Triton X-100, 100 mM EDTA, 10 mM Tris, pH 10). After decanting the lysis buffer, the slides were flooded with unwinding buffer (300 mM NaOH, 1 mM EDTA) and incubated at 4 °C for 20 min. The slides were then placed in an electrophoresis apparatus (CSL-COM20, Cleaver Scientific, Rugby, United Kingdom), and electrophoresis was performed in electrophoretic buffer (300 mM NaOH, 1 mM EDTA, pH > 13) for 20 min at 21 V and 29 mA. The slides were neutralized, dried overnight, and stained with 1 µg/mL 4′,6-diamidino-2-phenylindole (DAPI) for 60 min at 4 °C. Comet analysis was performed at 200× magnification using a fluorescence microscope (Nikon Corporation, Tokyo, Japan) equipped with a camera (Nikon Digital Sight DS-U3, Nikon Corporation, Tokyo, Japan) and coupled to Lucia Comet v. 7.0 software (Laboratory Imaging, Prague, Czech Republic). All samples were coded. Fifty images were randomly selected from each sample, and the percentage of DNA in the comet tail was measured as an indicator of DNA damage. The results were presented as mean ± SEM.

### 4.5. Metabolomic Analysis

For the purposes of sample coding and metabolomic data processing, the three experimental groups were assigned designations based on tissue type. The first letter indicated the dietary group. The second letter specified the tissue sampled: W denoted liver samples, and S denoted spleen samples. Consequently, the designations AW, BW, and CW referred to liver samples collected from groups A, B, and C, respectively, while AS, BS, and CS referred to spleen samples collected from groups A, B, and C, respectively. These codes were introduced solely to facilitate sample identification, statistical analysis, and figure preparation and they did not represent distinct experimental groups. The analyses were performed on three randomly selected samples of individual organs from each group. Three replicate measurements were carried out for each sample.

#### 4.5.1. Preparation of Extracts for LC-MS

Acetone (900 μL) and water (150 μL) were added to approximately 100 mg of tissue, and the mixture was vortexed for 1 min. The samples were homogenized three times for 60 s in a ball homogenizer using three steel balls (BeadBug 6; 4000 rpm, Sayreville, NJ, USA). The samples were then incubated at −20 °C for 20 min and centrifuged for 10 min at 4 °C and 12,000× *g*. The supernatants were transferred to Eppendorf tubes and dried under vacuum using a SpeedVac (0.2 mbar) for 24 h. The dry extracts were dissolved in methanol (100 mg of starting tissue per 100 μL of methanol), centrifuged again (4 °C, 12,000× *g*, 5 min), and transferred to HPLC vials with glass inserts.

#### 4.5.2. Quality-Control Procedure and Data Filtering

A pooled quality-control (QC) sample was prepared by combining equal aliquots of all study extracts and was analyzed repeatedly throughout each analytical sequence. In addition, a system-suitability standard mixture was analyzed at selected points of the sequence to monitor instrument performance. Analytical repeatability was assessed separately for the positive- and negative-ion datasets based on the pooled-QC injections.

Molecular features consistently detected in the pooled-QC samples were retained only when their RSD was below 30%. Among these features, 75.4% in the negative-ion dataset and 64.2% in the positive-ion dataset met the RSD < 30% criterion and were retained for subsequent statistical analyses. Samples within each ionization mode were analyzed in a single analytical batch. Therefore, no inter-batch correction was applied.

#### 4.5.3. UHPLC-QToF-MS and MS/MS Analysis of Extracts

Liquid chromatography–mass spectrometry analyses were performed using a Bruker Elute UHPLC system operated with Hystar 3.3 software and coupled to an ultrahigh resolution (>60,000) Bruker Impact II ESI–QTOF mass spectrometer (Bruker Daltonik GmbH, Bremen, Germany). Data were acquired and processed using DataAnalysis 4.2 (Bruker Daltonik GmbH, Bremen, Germany), TASQ (2022b), and MetaboScape (ver. 2022b) (Bruker Daltonik GmbH, Bremen, Germany). AutoMS/MS measurements were performed using a reverse-phase Bruker Intensity Solo C18 column (2 µm, 2.1 × 50 mm). Eluent A consisted of water with 0.1% HCOOH (Sigma-Aldrich, Louis, MO, USA), and eluent B consisted of acetonitrile (Sigma-Aldrich, St. Louis, MO, USA) with 0.1% HCOOH. A 5 μL aliquot of the extract was injected onto the column at a flow rate of 200 μL min^−1^ using 4% eluent B. The percentage of eluent B was changed over time as follows: 0 min, 1%; 0.56 min, 1%; 4.72 min, 99%; 5.56 min, 99%; 5.6 min, 1%; and 9.45 min, 1%. The solvent flow rate was 450 μL min^−1^, and column temperature was maintained at 40 °C. The column outlet was connected to the ESI source. AutoMS/MS measurements were conducted in both positive and negative ion modes in the *m*/*z* range of 50–1200. Collision-induced dissociation (CID) was performed with the following settings: an absolute area threshold of 5000 counts, active exclusion after 2 spectra, and release after 0.3 min. The isolation mass settings were as follows: width 4 for *m*/*z* = 100, width 5 for *m*/*z* = 300, width 6 for *m*/*z* = 500, and width 8 for *m*/*z* = 1000. The collision energy was set to 30 eV. Internal calibration on 10 mM sodium formate (Sigma-Aldrich, St. Louis, USA; water: isopropanol, 1:1, *v*/*v*) ions was performed automatically in Metaboscape using a syringe pump at an infusion flow rate of 1.5 µL/min in high-precision calibration (HPC) mode. Untargeted annotations were performed in MetaboScape (2022) using a mass deviation (Δm/z) threshold below 2 ppm and an *mSigma* value below 20 as the maximum acceptable deviations for the compound mass and isotopic pattern, respectively. Molecular formulas were generated using the Smart Formula tool with the following elements included: C, H, N, O, P, S, Cl, Br, I, and F. MS/MS spectra were automatically matched against the following MS/MS libraries: Bruker HMDB 2.0 (spectral data with retention times), MassBank of North America (MoNA, 2022), and NIST (Mass Spectrometry Data Center, 2022).

### 4.6. Statistical Analysis

Before parametric analyses, the data were checked for compliance with the assumptions of normality and equal variances. The distribution of residuals was tested using the Shapiro–Wilk test, and the homogeneity of variance was assessed using Levene’s test. As neither test indicated a violation of assumptions (*p* > 0.05), subsequent analyses were performed using repeated-measures ANOVA, with the study group serving as the independent factor. The parameters measured in rats were treated as dependent variables. When significant main effects were detected, post hoc comparisons were carried out using Tukey’s HSD test. All statistical analyses, including descriptive statistics, were performed using Statistica version 13.3 (TIBCO Software Inc., Palo Alto, CA, USA).

Linear regression analysis with interaction terms was used to determine whether the relationship between the concentration of an individual SCFA (X) and the pool of the remaining SCFAs differed between the study groups (A, B, C; *n* = 15 in each group). To avoid a part-whole artifact, the sum of the remaining acids was used as the dependent variable for each SCFA (Total − X). A separate model was fitted for each SCFA:

$$Y=\beta_{0}+\beta_{1}X+\beta_{2}I(A)+\beta_{3}I(B)+\beta_{4}(X\cdot I(A))+\beta_{5}(X\cdot I(B))+\varepsilon$$

where group C was the reference category. In this approach, β_1_ describes the slope (X–Y relationship) in group C, whereas β_4_ and β_5_ represent the differences in slopes for groups A and B, respectively, relative to group C. The significance of slope differences was assessed using a joint test for the interaction terms (β_4_ = β_5_ = 0; F-test, df = 2) and, additionally, tests for the individual contrasts A vs. C and B vs. C.

To evaluate the relationships between the study groups, SCFA concentrations, fecal enzyme activity, and genotoxicity, multivariable linear regression analyses were performed separately for each analyzed time point. In each model, genotoxicity was treated as the dependent variable. Group was included as a categorical predictor, whereas formic, acetic, propionic, butyric, isobutyric, isovaleric, and valeric acid concentrations, β-glucuronidase activity, and β-glucosidase activity were included as continuous predictors.

All metabolite datasets were exported using Metaboscape ver. 2022b, and then examined using MetaboAnalyst 6.0 [114]. The data were log-transformed and autoscaled prior to unsupervised principal component analysis (PCA) and supervised orthogonal partial least squares discriminant analysis (OPLS-DA). The models were validated using 5-fold cross-validation, and model significance was assessed by permutation testing. The following criteria were applied to identify the metabolites that most significantly distinguished the two groups: VIP > 1, *p* < 0.05, FDR < 0.05, and fold-change (FC) > 1.2 or < 0.83. Pathway enrichment analyses were conducted in MetaboAnalyst 6.0, using the Small Molecule Pathway Database (SMPDB) to identify the major chemical classes of discriminating metabolites and the Kyoto Encyclopedia of Genes and Genomes (KEGG) database with *Rattus norvegicus* as the reference organism for pathway mapping.

## 5. Conclusions

A diet containing 35% *T. molitor* meal induced concurrent diet-associated differences in gut microbial functional activity and host metabolism in rats. The insect-based diet altered colonic fermentation patterns, as reflected by reduced fecal SCFA concentrations and higher β-glucosidase and β-glucuronidase activities than in the comparison groups. Fecal water genotoxicity was significantly reduced in the *T. molitor* group compared with the group fed the chicken hydrolysate diet.

The untargeted metabolomic analysis revealed diet-dependent differences in liver and spleen metabolite profiles, primarily involving metabolites assigned to lipid-, bile acid-, nucleotide-, and amino acid-related pathways. These findings identify hepatic and splenic metabolic pathways associated with the dietary treatments but do not demonstrate their functional activation or direct effects on immune function.

These findings lay the groundwork for future studies focused on linking metabolomic alterations with physiological and performance outcomes. However, the decrease in butyrate production raises potential concerns for colonic health and warrants further investigation. As this study did not directly assess intestinal histology or inflammatory responses, the biological consequences of these changes for gut health remain unclear. Future research should investigate the interactions between insect-based diets and dietary fibers such as resistant starch, as well as their long-term effects on gut health and systemic metabolism.

## Figures and Tables

**Table 1:** Concentrations of short-chain fatty acids (SCFAs) in fecal samples collected from rats fed different diets.

SCFA	Time	Group A (Mean ± SD)	Group B (Mean ± SD)	Group C (Mean ± SD)
**Formic Acid**	Week 4	11.71 ± 6.60^a^	24.02 ± 6.11^b^	19.48 ± 10.37^b^
	Week 6	12.78 ± 6.12^a^	28.68 ± 15.21^b^	20.63 ± 8.35^ab^
	Week 8	17.17 ± 8.79 ^a^	39.36 ± 13.64^b^	31.12 ± 25.66 ^ab^
**Acetic Acid**	Week 4	12.17 ± 7.01^a^	31.58 ± 8.89^b^	15.85 ± 4.06^a^
	Week 6	9.76 ± 5.15^a^	32.45 ± 6.54^b^	24.90 ± 22.36^b^
	Week 8	15.97 ± 8.82^a^	49.03 ± 16.06^b^	31.92 ± 39.79^ab^
**Propionic Acid**	Week 4	8.27 ± 4.87^a^	13.58 ± 4.09^b^	9.16 ± 3.67^a^
	Week 6	7.26 ± 4.63^a^	12.87 ± 2.88^a^	16.04 ± 21.00^a^
	Week 8	15.69 ± 19.37^a^	20.60 ± 10.25^a^	23.43 ± 41.88^a^
**Butyric Acid**	Week 4	11.38 ± 2.94^†d^	15.75 ± 4.07^†bc^	19.18 ± 4.95^c^
	Week 6	14.72 ± 6.59^†d^	39.19 ± 18.16^†bc^	50.79 ± 32.75^†c^
	Week 8	26.06 ± 15.35^†c^	95.57 ± 58.96^†a^	66.43 ± 33.56^†ab^
**Isobutyric Acid**	Week 4	3.41 ± 3.29^a^	15.83 ± 6.55^a^	7.28 ± 4.73^a^
	Week 6	2.63 ± 3.03^a^	10.83 ± 5.87^a^	43.73 ± 109.75^a^
	Week 8	5.31 ± 3.48^a^	20.47 ± 14.28^a^	19.85 ± 46.26^a^
**Isovaleric Acid**	Week 4	8.72 ± 5.66^a^	18.11 ± 7.44^b^	18.27 ± 7.92^b^
	Week 6	6.10 ± 4.34^a^	13.08 ± 6.18^a^	42.27 ± 47.45^b^
	Week 8	11.46 ± 7.20^a^	43.48 ± 54.67^a^	34.43 ± 51.34^a^
**Valeric Acid**	Week 4	5.86 ± 4.30^a^	26.29 ± 13.59^b^	18.44 ± 7.44^b^
	Week 6	6.51 ± 3.41^a^	28.34 ± 9.09^b^	32.36 ± 37.54^b^
	Week 8	8.16 ± 7.76^a^	44.52 ± 46.57^a^	35.95 ± 63.24^a^

Legend: Groups: A (35% *T. molitor* meal), B (chicken hydrolysate diet), C (standard laboratory rat diet). Letters a–d denote homogeneous groups for individual measurement days analyzed separately. ^†^ Statistically significant group vs. time interactions were observed.

## Data Availability

The original contributions presented in this study are included in the article/Supplementary Material. Further inquiries can be directed to the corresponding author.

## Supplementary Materials

The following supporting information can be downloaded at: https://www.mdpi.com/article/10.3390/ijms27177627/s1.
