# Supplementary material for: Integrated Assessment of the Functional Activity of the Gut Environment and Metabolic Profiles in Rats Fed an Insect-Based (Tenebrio molitor) Diet
Source: Int J Mol Sci. 2026 Aug 26;27(17):7627. doi: 10.3390/ijms27177627 (PMC13566692; doi:10.3390/ijms27177627)
Supplement: Supplementary file 1 [file ijms-27-07627-s001.zip › Table S23.pdf]

Table S23. Proximate composition of the standard laboratory diet and experimental diets containing chicken hydrolysate and 35% *Tenebrio molitor* diet.

Dietary composition	Standard laboratory rat diet	Chicken hydrolysate diet	35%*Tenebrio molitor* diet
Crude Protein (%)	21.47 ± 1.78	25.93 ± 0.91	25.83 ± 0.78
Crude Fat (%)	6.01 ± 0.41	13.51 ± 0.74	13.79 ± 0.65
Crude Ash (%)	4.58 ± 0.49	5.68 ± 0.67	5.37 ± 0.43
Crude Fiber (%)	5.01 ± 0.46	5.26 ± 0.54	5.22 ± 0.49
Carbohydrates (%)	62.93 ± 2.17	49.62 ± 1.75	49.79 ± 1.04
Estimated energy value (kcal/100 g)	391.7	423.8	426.6
Alanine (% of quantified amino acids)	6.57 ± 0.27	6.12 ± 0.07	7.71 ± 0.07
Arginine (% of quantified amino acids)	6.26 ± 0.12	7.04 ± 0.17	4.72 ± 0.33
Cystine (% of quantified amino acids)	1.65 ± 0.08	2.54 ± 0.06	4.91 ± 0.33
Phenylalanine (% of quantified amino acids)	4.87 ± 0.10	4.77 ± 0.08	5.51 ± 0.16
Glycine (% of quantified amino acids)	5.38 ± 0.09	7.55 ± 0.10	5.84 ± 0.23
Histidine (% of quantified amino acids)	2.01 ± 0.10	2.28 ± 0.11	2.11 ± 0.10
Aspartic acid (% of quantified amino acids)	12.50 ± 0.16	9.56 ± 0.09	9.41 ± 0.92
Glutamic acid (% of quantified amino acids)	19.16 ± 0.23	16.50 ± 0.06	18.52 ± 1.16
Leucine (% of quantified amino acids)	8.33 ± 0.14	7.93 ± 0.05	7.63 ± 0.18
Lysine (% of quantified amino acids)	6.75 ± 0.10	5.83 ± 0.11	4.05 ± 0.35
Methionine (% of quantified amino acids)	1.98 ± 0.09	3.16 ± 0.17	1.46 ± 0.11
Proline (% of quantified amino acids)	6.55 ± 0.04	5.91 ± 0.04	6.48 ± 0.13

Serine (% of quantified amino acids)	5.30 ± 0.06	5.32 ± 0.16	4.30 ± 0.22
Threonine (% of quantified amino acids)	4.31 ± 0.03	4.91 ± 0.05	3.94 ± 0.21
Tyrosine (% of quantified amino acids)	3.63 ± 0.07	4.42 ± 0.07	5.48 ± 0.18
Valine (% of quantified amino acids)	4.75 ± 0.05	6.17 ± 0.08	7.93 ± 0.01
Vitamin A (IU/kg)*	15000	18000	18000
Vitamin D_3 (IU/kg)*	1000	1500	1500
Vitamin E (mg/kg)*	90	500	500
Biotin (µg/kg)*	400	100	100
Saturated fatty acids (% of quantified fatty acids)	NA	32.02 ± 0.61	24.64 ± 1.00
Monounsaturated fatty acids (% of quantified fatty acids)	NA	33.33 ± 2.25	39.01 ± 1.70
Omega-6 polyunsaturated fatty acids (% of quantified fatty acids)	NA	28.11 ± 1.64	31.24 ± 1.29
Omega-3 polyunsaturated fatty acids (% of quantified fatty acids)	NA	6.54 ± 0.84	5.11 ± 0.37

Legend: *-declared; NA- data not available.

The complete diet formulations are protected under patent No. 247885—hypoallergenic dog food. The patent covers a hypoallergenic feed formulation for dogs suffering from food-responsive enteropathies. Hypoallergenic dog food contains *T. molitor* meal (inclusion level: 10% to 60%, preferably 35%), potato and/or sweet potato by-products (10% to 65%, preferably 48%), plant substrates of the plantain family (0.01% to 15%, preferably 7.3%), animal or vegetable fat (0.1–15%), herbs and minerals (0.01% to 5%, preferably 1.8%), berries (0.01% to 8%, preferably 0.6%), functional additives such as water- and fat-soluble vitamins (0.01% to 15%, preferably 6.3%), and plants of the flax family (0.01% to 5%, preferably 1%). The patented formulation has the following nutritional composition: crude protein—11% to 41%, (preferably 25%); crude fat—3% to 27%, (preferably 15%); crude ash—1% to 9%; (preferably 5%); and crude fiber—1% to 9%, (preferably 5%). The diets were originally developed as hypoallergenic options for dogs with food-responsive enteropathies.

In the reference diet for Group B, insect meal was replaced with a commercially available chicken hydrolysate, and the formulation was subsequently nutritionally balanced using fish oil and thermally processed potato by-products. The quantities of protein, lipid, and fiber sources were adjusted to match the nutritional profile of the chicken hydrolysate, ensuring a nutritional composition comparable to that of the insect-based formulation. The chicken hydrolysate diet was selected as a practical and industrially relevant reference formulation, particularly for feeding companion animals with food-responsive enteropathies. Apart from adjustments

required for the experimental protein source and nutritional balancing, the same general formulation framework was applied to both experimental diets.

Selected technological and formulation details, including precise ingredient proportions and some aspects of nutritional balancing, constitute the subcontractor’s intellectual property and were not available for disclosure. According to the information provided by the subcontractor, both experimental diets were manufactured using industrial dry-feed extrusion. Following extrusion, the feed granules were dried at approximately 105 °C for approximately 16 min and subsequently cooled for approximately 6 min to obtain a shelf-stable dry product.

The precise composition and manufacturing processes of the diet for Group C constitute the manufacturer's intellectual property. According to available information, the feed consists of: cereal grains and their derivatives, soybean meal, linseed meal, feed-grade yeast, potato protein, dried whey, macro- and micro-elements, and vitamins.
