# Supplementary material for: Integrated Assessment of the Functional Activity of the Gut Environment and Metabolic Profiles in Rats Fed an Insect-Based (Tenebrio molitor) Diet
Source: Int J Mol Sci. 2026 Aug 26;27(17):7627. doi: 10.3390/ijms27177627 (PMC13566692; doi:10.3390/ijms27177627)
Supplement: Supplementary file 1 [file ijms-27-07627-s001.zip › Table S24.pdf]

Table S24a. Changes in body weight of rats fed diets containing 35% *Tenebrio molitor* meal (Group A), chicken hydrolysate (Group B), or standard laboratory feed (Group C) during the experimental period.

**Time**	**Rat body weight (g)**					
	Group A		Group B		Group C	
	Mean	SD	Mean	SD	Mean	SD
Start	265.07	9.61	267.87	8.88	269.27	7.29
Week 1	259.13	10.36	260.73	8.86	267.20	7.88
Week 2	264.00	8.76	266.07	8.33	269.07	8.87
Week 3	266.53	9.26	268.53	8.93	269.73	9.49
Week 4	266.07	9.23	269.40	9.19	269.93	9.54
Week 5	266.53	8.35	269.00	9.54	268.60	10.04
Week 6	268.47	9.79	269.80	8.44	270.07	9.35
Week 7	268.67	6.48	271.20	8.51	269.33	10.11
End	268.33	8.65	271.60	8.60	270.40	8.55

Table S24b. Weekly feed intake of rats fed diets containing 35% *Tenebrio molitor* meal (Group A), chicken hydrolysate (Group B), or standard laboratory feed (Group C) during the experimental period.

**Time**	**Weekly feed intake (g)**					
	Group A		Group B		Group C	
	Mean	SD	Mean	SD	Mean	SD
Week 1	88.80	1.57	88.53	1.92	115.40	2.69
Week 2	95.73	1.49	95.00	1.81	117.20	1.42
Week 3	97.47	0.83	97.00	1.51	118.07	1.33
Week 4	97.47	1.25	97.27	1.58	117.87	1.77
Week 5	97.87	0.35	97.87	0.52	118.87	0.52
Week 6	97.67	0.90	97.67	0.90	118.67	0.82
Week 7	97.80	0.56	97.93	0.26	118.47	1.13
Week 8	97.40	1.12	96.80	1.37	118.67	0.90
Week 9^	55.67	0.82	55.60	0.74	67.67	0.72

Legend: ^— Values represent cumulative feed intake per rat during the final 4 days of the experiment.
